# Evaluation of an Affinity-Enhanced Anti-SARS-CoV2 Nanobody Design Workflow Using Machine Learning and Molecular Dynamics

**DOI:** 10.1021/acs.jcim.4c01023

**Published:** 2024-10-02

**Authors:** Zsolt Fazekas, Dóra Nagy-Fazekas, Boglárka Mária Shilling-Tóth, Péter Ecsédi, Pál Stráner, László Nyitray, András Perczel

**Affiliations:** † Medicinal Chemistry Research Group, HUN-REN Research Centre for Natural Sciences, Magyar Tudósok Körútja 2, H-1117 Budapest, Hungary; ‡ HUN-REN-ELTE Protein Modeling Research Group, Hungarian Research Network (HUN-REN), Institute of Chemistry, Eötvös Loránd University, Pázmány Péter sétány 1/A, Budapest H-1117, Hungary; § Hevesy György PhD School of Chemistry, Institute of Chemistry, Eötvös Loránd University, Budapest, Pázmány Péter sétány. 1/A, Budapest H-1117, Hungary; ∥ Laboratory of Structural Chemistry and Biology, Institute of Chemistry, Eötvös Loránd University, Pázmány Péter sétány 1/A, Budapest H-1117, Hungary; ⊥ Department of Biochemistry, Eötvös Loránd University, Pázmány Péter sétány 1/C, Budapest H-1117, Hungary

## Abstract

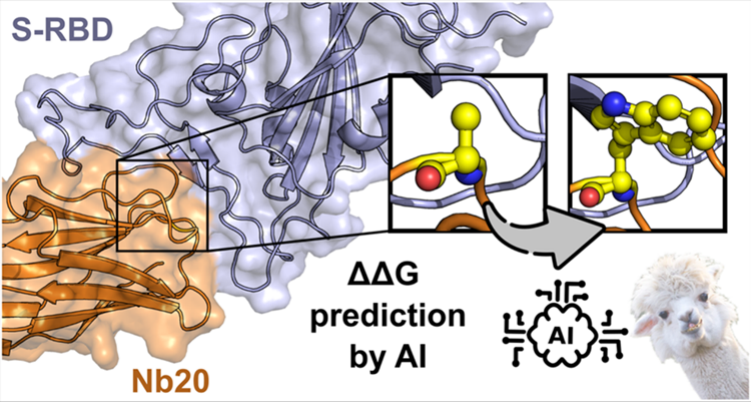

*In silico* optimization of protein binding has received a great deal of attention in the recent years. Since *in silico* prefiltering of strong binders is fast and cheap compared to *in vitro* library screening methods, the advent of powerful hardware and advanced machine learning algorithms has made this strategy more accessible and preferred. These advances have already impacted the global response to pandemic threats. In this study, we proposed and tested a workflow for designing nanobodies targeting the SARS-CoV-2 spike protein receptor binding domain (S-RBD) using machine learning techniques complemented by molecular dynamics simulations. We evaluated the feasibility of this workflow using a test set of 3 different nanobodies and 2 different S-RBD variants, from *in silico* design and bacterial expression to binding assays of the designed nanobody mutants. We successfully designed nanobodies that were subsequently tested against both the wild-type (Wuhan type) and the delta variant S-RBD and found 2 of them to be stronger binders compared to the wild-type nanobody. We use this case study to describe both the strengths and weaknesses of this *in silico* assisted nanobody design strategy.

## Introduction

The severe acute respiratory syndrome coronavirus 2 (SARS-CoV-2) pandemic that began in 2019, has become one of the most significant global health crises in the recent history. The virus responsible for the pandemic causes the coronavirus disease 2019 (COVID-19), with symptoms varying greatly between unobservable- (asymptomatic), mild- (e.g., fever, cough, loss of smell), and severe illness (low SpO2, respiratory failure, organ dysfunction).^1^ The outbreak spread rapidly around the world, often accompanied by bacterial coinfections,^2^ resulting in a devastating loss of life. As the disease became more widespread, research into prevention and treatment options intensified. Although vaccines were developed by several companies (e.g., Johnson & Johnson, Moderna, Pfizer, Novavax),^3^ the treatment of those already infected with severe symptoms relied on intensive hospital care, often with the help of invasive mechanical ventilation.^4^

To mitigate the effects of the virus, interest in SARS-CoV-2 antibodies and antibody mimetics has increased, leading to the development of countless alternatives that are collected and organized in the Coronavirus Antibody Database.^5^ Protein-based treatments have the advantage over small-molecule options that they are highly specific, cause fewer side effects and are better tolerated. Furthermore, the FDA also favors protein therapeutics with faster approval times.^6^ Viral surface accessibility limits the possible target proteins for SARS-CoV-2 variants to the small set of spike, membrane, and envelope proteins and explains why the majority of antibodies bind to the most easily accessible spike receptor binding domain (S-RBD). This domain is located on the “tip” of the homotrimeric spike protein and is responsible for the binding to the human angiotensin converting enzyme 2 (ACE2) in the initiating step of host cell invasion. The S-RBD fluctuates between up- (or open-) and down- (or closed-) conformers,^7,8^ allowing it to contact its ACE2 partner in the open conformation, and to partially evade an immune response in its closed form.

Nanobodies, also called single-chain antibodies, are a class of engineered proteins that combine the specific partner recognition ability of antibodies with a small and simple, single-domain structure. A subclass of nanobodies, called V_H_H fragments, are engineered from heavy chain-only camelid antibodies through the omission of the constant domains. The simplicity and strong binding ability of V_H_Hs make them excellent agents for anti-SARS-CoV-2 development. Several S-RBD targeting nanobodies have been successfully developed, such as H11-H4,^9^ SR4,^10^ MR17,^10^ Nb20,^11^ Nb6,^12^ and ab8.^13^ Furthermore, the VHH-72 nanobody that was developed against SARS-CoV-1, was found to be cross-reactive against SARS-CoV-2,^14^ albeit with reduced affinity. We have previously introduced a protocol for the *Escherichia coli*-based bacterial expression of both nanobodies and the S-RBD of SARS-CoV-2, using a fusion construct containing a periplasmic translocator protein called Ecotin.^15^ Using this approach allows the efficient production of large quantities of recombinant S-RBD proteins. In light of this, we envisioned an *in vitro* SARS-CoV-2 S-RBD targeting nanobody optimization platform, where production of both the S-RBD variants and the nanobody mutants are performed by bacterial expression.

To limit the expression to potentially optimized binders, *in silico* methods can be used to filter out irrelevant variants from the high-dimensional mutation space. An important descriptor of anti- and nanobodies is the Gibbs free energy change associated with binding to its antigen partner per mole (Δ*G*). Stronger binding means more negative values, and a Δ*G* above the 0 kcal/mol threshold indicates that the equilibrium is shifted toward the monomers. While Δ*G* assesses the binding strength of two proteins, ΔΔ*G* = Δ*G*_mut_ – Δ*G*_WT_ is used to assess the quantitative effects of mutation(s). ΔΔ*G* describes the stabilizing/destabilizing effect of the change from a wild-type (WT) protein to its mutant variant (mut). *In vitro* techniques to measure these values include biolayer interferometry (BLI), surface plasmon resonance (SPR), isothermal titration calorimetry (ITC), or nuclear magnetic resonance (NMR) spectroscopy. One of the challenges in computational biochemistry is to accurately predict Δ*G* and ΔΔ*G* without the need for such techniques. A computationally inexpensive approach to approximating ΔΔ*G* is to use the WT complex structure along with the mutation queries as inputs to machine learning (ML) models. This led to the development of algorithms such as iSEE,^16^ mCSM-PPI2,^17^ mCSM-AB2,^18^ TopNetTree,^19^ MutaBind2,^20^ SAAMBE-3D,^21^ GeoPPI.^22^ A detailed review can be found in the reference.^23^ Although these methods are fast and easy to use, they are not fail-safe for every target.

Another, more classical approach is to use molecular dynamics (MD) simulations for the same purpose. Such simulations aim to reproduce *in silico* the allowed conformers of a protein (or protein complex). The result of these simulations are trajectories, a series of time-correlated protein states (or “frames”). In the case of MD, thermodynamic integration, free energy perturbation, or umbrella sampling provide the most accurate estimates of ΔΔ*G*. Despite the accuracy of these methods, they are rarely used techniques due to their high computational cost. Instead, end point methods are often used, such as the molecular mechanics with Poisson–Boltzmann and surface area solvation method (MM/PBSA) and generalized Born and surface area solvation method (MM/GBSA).^24^ Although less accurate, end-point methods require less computational time and resources. As their names suggest, they consider only the end-states of the system (e.g., uncomplexed and complexed states). The approximated Gibbs free energy change is either calculated from one (only the complex is simulated), two (the complex and one partner are simulated), or three (the complex and both partners are simulated) MD trajectories, depending on the desired accuracy (3 > 2 > 1) and precision (3 < 2 < 1).

**Figure 1:**
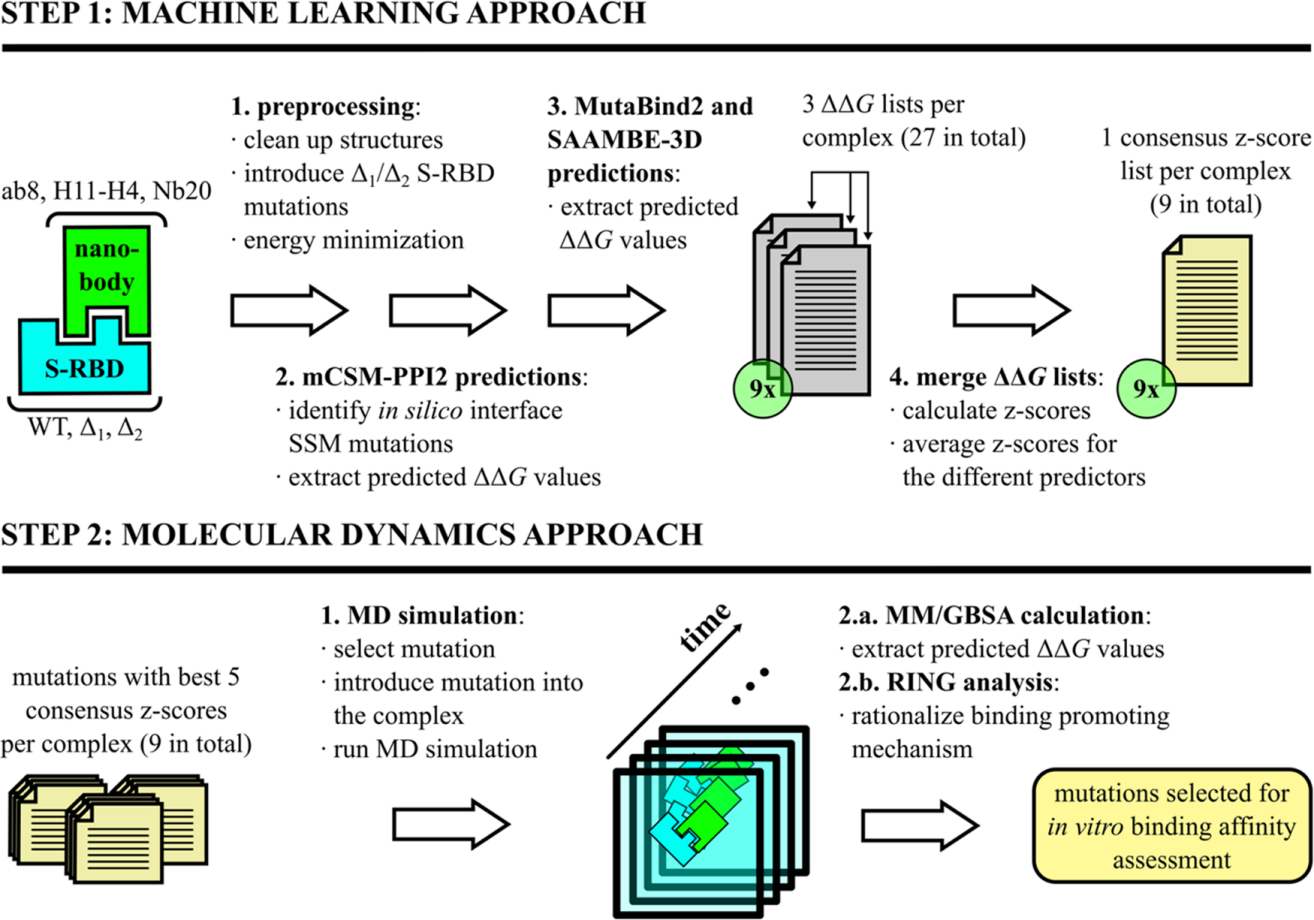
Workflow diagram for the tested ML and MD approaches. The upper track illustrates the structure preprocessing steps (which result in the PDB files inputted into the three ML models), the mutation list selection by the mCSM-PPI2 Web server’s “interface analysis” function, the ML runs, and the prediction list postprocessing steps. The bottom track shows the MD simulation and trajectory analysis steps, namely the MM/GBSA calculations and the RING residue–residue interaction detection.

In this work, we aim to investigate the applicability of a ML and MM/GBSA workflow for the design of novel, specific anti-S-RBD nanobodies (Figure 1). Nanobody constructs were generated against both the WT, i.e., Wuhan, and the Delta B.1.617.2 variants. We used the consensus of three different ML methods, namely mCSM-PPI2, MutaBind2 and SAAMBE-3D, to prefilter mutations in the case of nanobodies H11-H4, Nb20, and ab8. These predictors were selected due to their high reported accuracy (based on correlation and error), availability, ease of use, and different model selection approaches (extra trees, random forest, and XGBoost, respectively). Mutations with the best consensus scores were then subjected to MD simulations and MM/GBSA calculations, in order to further validate their binding promoting effects and to understand the mechanism behind these effects at the molecular level. Finally, mutations were selected for expression in *E. coli* and their affinity to the two S-RBDs was measured using biolayer interferometry (BLI). The proposed workflow could support and/or partially replace time-consuming and costly methods in the future, such as immunization or phage display.

## Materials and Methods

### ML Predictions, MD Simulations, and MM/GBSA Calculations

Structures with PDB IDs of 6ZBP (H11-H4), 7JVB (Nb20), 7MJI (ab8), and 6ZDH (full spike protein) were downloaded from www.rcsb.org. To alleviate any differences between the nanobody/S-RBD complexes, such as chain breaks and conformational differences far from the interface, a single S-RBD model was created using residues 333–529 of the 6ZDH structure and was used to replace the complexing S-RBD within the nanobody complexes. The 6ZDH S-RBD was aligned with each of the S-RBDs within the complex structures using PyMol^25^ and then substituted for the original. Then, the Optimize command of FoldX^26^ was used to repair side chains and remove van der Waals collisions, followed by a steepest descent energy minimization step with the GROMACS 2021.4 software.^27^ The convergence criteria for the minimization were a maximum force of 50 kJ × mol^–1^ × nm^–1^ and a maximum number of steps of 11,000.

Mutations to these initial structures were also introduced using FoldX with the BuildModel protocol. First, the delta variant S-RBD structures were generated. Two subvariants were investigated, subvariant Δ_1_ (B.1.617.2) with mutations L452R, T478 K and subvariant Δ_2_ (B.1.617.2+K417N) with an additional mutation of K417N.^28^ After introducing these mutations, the structures were energy minimized again.

*In silico* site saturation mutagenesis (SSM) studies were then performed on the nanobodies in search of ΔΔ*G* values using three freely available machine learning Web servers: mCSM-PPI2,^17^ SAAMBE-3D,^21^ and MutaBind2.^20^ Interface residues were determined by mCSM-PPI2 using the Interface Analysis option and then custom Python scripts were used to convert the mCSM-PPI2 output format into SAAMBE-3D and MutaBind2 input formats. After collecting all SSM results, which are mutation-ΔΔ*G* pair lists for each initial structure (WT S-RBD, Δ_1_ and Δ_2_ complexes), nanobody mutation ΔΔ*G* values were merged. Lists (***L***) from the different Web servers were first standardized by subtracting the median ΔΔ*G* from each value, and then dividing the difference by the median absolute deviation (MAD), resulting in the so-called *z*-scores
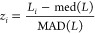
These *z*-scores were then simply averaged to get the consensus *z*-score
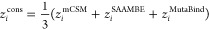
This procedure is similar to the prediction-unifying technique described previously.^29^ Nanobody mutations were then ranked by their consensus *z*-scores, resulting in the list from which mutations were selected.

MD simulations were carried out using GROMACS. Complexes were solvated using the tip4p water model,^30^ while sodium and chloride ions were added to electrostatically neutralize the system (final concentration of NaCl is 0.15 M). The AMBER-ff99SBildnp-star^31^ force field was used for all runs. The following steps were used to equilibrate the proteins: first, a steepest descent integrator with position restraints of 1000, 500, 100 and then 0 kJ × mol^–1^ × nm^–2^ and a maximal force tolerance of 50 kJ × mol^–1^ × nm^–2^ was used. Next, an NVT equilibration was performed using the leapfrog integrator for 50,000 steps with a step size of 2 fs with positional restraints of 1000, 500, 100, and 0 kJ × mol^–1^ × nm^–2^ at 310 K. Finally, an unconstrained NPT step was followed to allow the introduction of pressure. Complexes were simulated for total simulation times between 331 and 1000 ns.

After centralizing the resulting trajectories, the gmx_MMPBSA software^32^ was used to perform single-trajectory MM/GBSA calculations. The trajectories were analyzed from 50 ns to their ends with an interval of 100 ps. The leaprc.ff99SBildn force field was used with GB-OBC1 Generalized Born Method^33^ and 0.15 M salt concentration. No entropy calculations were performed, generating ΔΔ*H* output values.

### Construction of Expression Plasmids

Cloning was carried out similarly as in previous studies.^15^ First, a modified pMAL plasmid was generated by complementing the original vector with a thrombin cleavable N-terminal Ecotin-His_6_ tag, a method that has been shown to facilitate nanobody expression in bacterial systems. A pET-32b derived multiple cloning site was introduced to facilitate restriction cloning of nanobody constructs. Next, the genes of each target mutant nanobody were synthesized as a double-stranded gene fragment (minigene) (IDT) and were ligated into the vector (*Bam*HI-XhoI restriction enzyme pair was used). See SText1–8 for further details.

DH5α competent cells (Subcloning Efficiency DH5α competent cells ThermoFisher, cat. No. 18265017) were used for plasmid cloning procedures and transformed by heat shock transformation, following the manufacturer’s instructions. Transformed cells were then selected on LB agar containing ampicillin (AMP) at a concentration of 100 μg/L.

### Nanobody Expression and Purification

Similarly, ecotin fusion proteins were overexpressed in *E. coli* Shuffle-T7 cells, as described in the reference.^15^ Cells were grown overnight at 37 °C on LB agar plates containing AMP (100 μg/mL). Single colonies were transformed to 50 mL LB (100 μg/mL AMP) and were incubated for 3 h at 37 °C with shaking at 180 rpm. For protein production, these precultures were inoculated (100-fold) into 2YT medium containing 2 g/L glucose, and cells were grown at 37 °C until the absorbance of the medium at 600 nm reached 0.8 (OD600). Cells were induced by adding 0.5 mM isopropyl β-d-1-thiogalactopyranoside (IPTG). Expressions were carried out overnight (∼12 h) at 30 °C. Cultures were harvested by centrifugation (4500 g for 10 min at 4 °C). The pelleted cells were resuspended in 50 mL buffer A (300 mM NaCl, 50 mM Na_2_HPO_4_, pH = 8.0) per L of medium and cells were disrupted by ultrasonication. After centrifugation (23,000*g*, 20 min, 4 ° C) the supernatants were collected and applied to a 5 mL HisTrap column at a flow rate of 0.8 mL/min. After binding, the column was washed with buffer A. Elutions were performed with an isocratic gradient using an elution buffer (buffer A complemented with 250 mM imidazole) at a flow rate of 4 mL/min.

The collected fractions were pooled and dialyzed in 4 L PBS. After dialysis, the fusion protein was cleaved by thrombin protease (0.01 μL/μg) overnight. The constructs were applied to Ni^2+^ column again, but this time the flow-through fractions were collected. The samples were then concentrated using a 10 kDa ultrafiltration device and were further purified with size exclusion chromatography (Superdex 75 10/300 GL column) (SFigure 1–6). The fractions from the different purification steps were analyzed by SDS-PAGE to determine the correct molecular weight (SFigure 7–8). Protein samples dissolved in PBS were stored at −80 °C until further use. Expression yields are indicated in STable1.

### RBD Domain Expression and Purification

The His-tagged Spike RBD variants were expressed in Chinese Hamster Ovary (CHO) Express cells in the ExpiCHO expression system for 12 days at 34 °C by 8% CO_2_ containing CO_2_ thermostat according to the manufacturer’s instructions. Corresponding DNA sequence data can be read in SText1–2. The expressed protein was deposited into the ExpiCHO cell medium, which was collected and purified by using Ni^2+^ immobilized metal affinity chromatography (Ni-IMAC by HisTrap Cytiva column, Product no:10316910) using the FPLC Akta Pure 2 system. After purification the sample was then dialyzed in PBS buffer (ExpiCHO Expression System User Guide, Pub. No. MAN0014337 Rev. D.0).

### Biolayer Interferometry Measurements

The S-RBD/nanobody interactions were measured on Anti-Penta-His biosensor tips managed by an Octet K2 (Sartorius) system. Proteins were dialyzed in PBS to minimize signal errors caused by the buffer difference. Note that 0.02% Tween-20 was also used here to prevent aspecific associations of molecules. Sensors were loaded with a 5 μM S-RBD solution. Associations were followed by adding nanobodies at different concentrations. A solution of 10 mM glycine at a pH of 2.5 was used to regenerate the sensor tips after each measurement. The association and dissociation curves were analyzed using the Octet System software and visualized with Python scripts using the Matplotlib^34^ package.

## Results and Discussion

### Application of ML Methods

**Figure 2:**
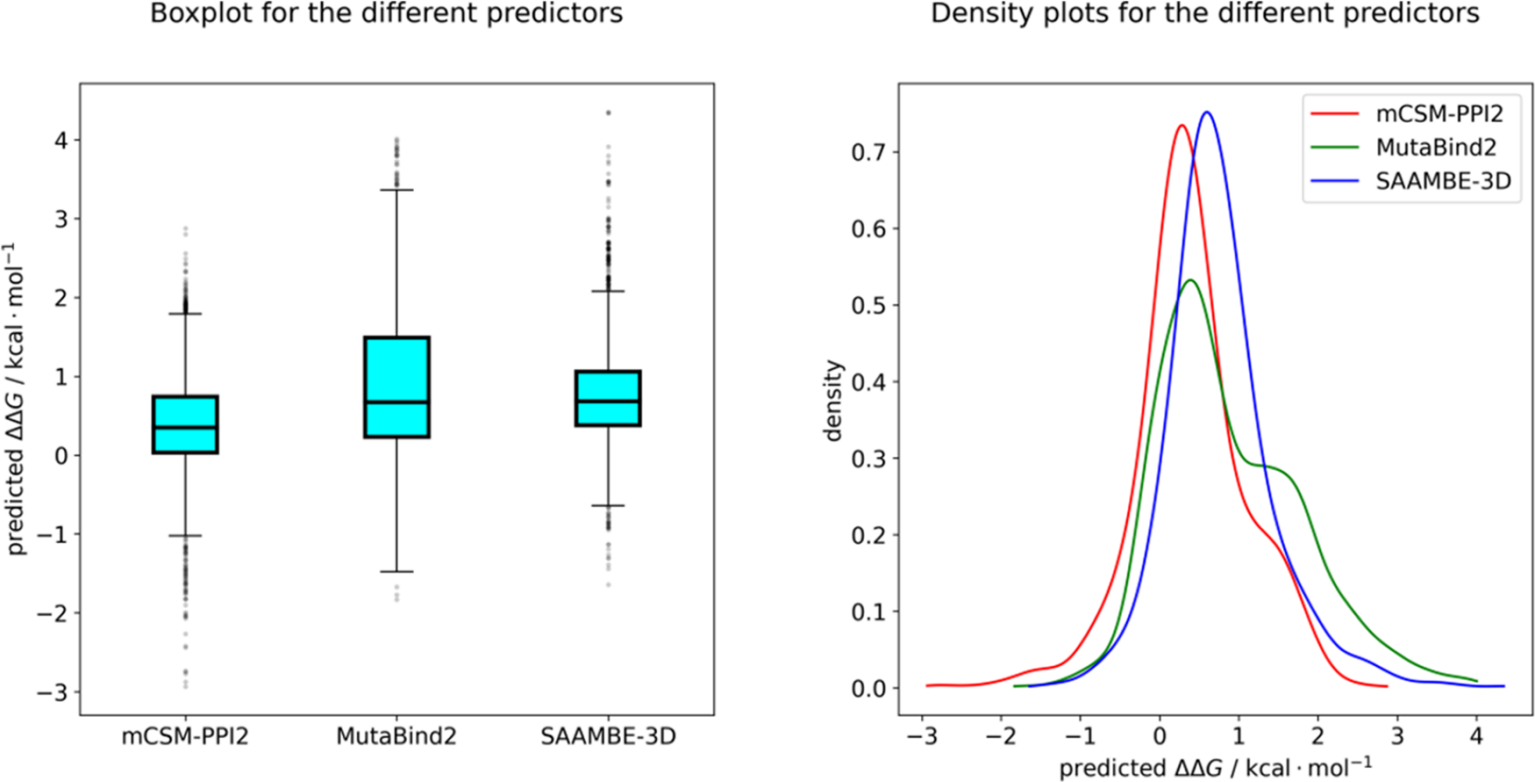
(A) Box-and-whiskers plots for the predicted ΔΔ*G* values using different ML methods collected for all three nanobodies and S-RBD variants. The middle line indicates the median of the dataset, boxes extend from the lower to the upper quartile, and the whiskers indicate the data range. Outliers are indicated by partially opaque dots. (B) Kernel density estimation of the predicted ΔΔ*G* values resulting from the different ML methods.

**Figure 3:**
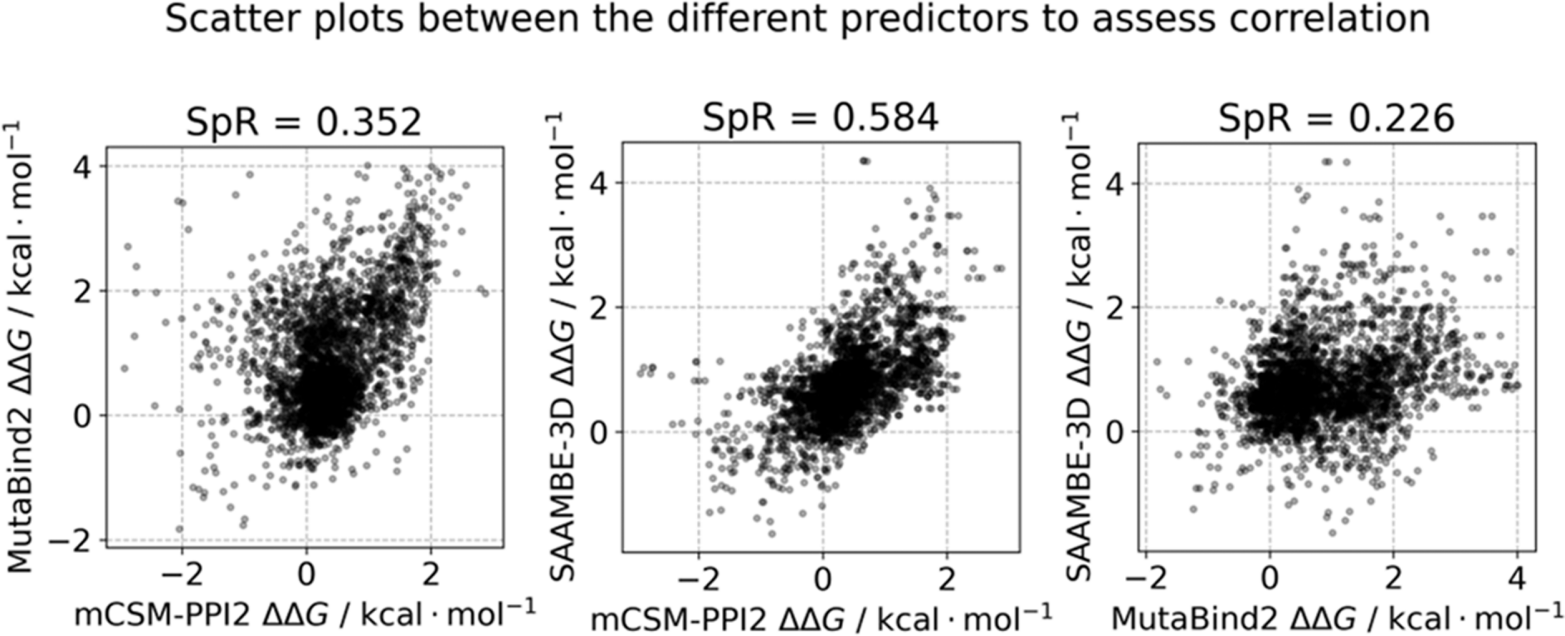
Cross-scatter plots between the results of the different predictors to visualize predictor correlations.

In the first step of our study, mCSM-PPI2, SAAMBE-3D and MutaBind2 were used to perform *in silico* site saturation mutagenesis (SSM) to scan all mutations on the S-RBD/nanobody interaction surface. The distribution of the predicted mutant ΔΔ*G* values was analyzed (Figure 2) using statistical analysis techniques. 77.5, 91.8, and 84.9% of the mutation data points are predicted to be complex destabilizing, according to the mCSM-PPI2, SAAMBE-3D and MutaBind2 protocols, respectively (Figure 2A and Table 1). To assess the correlation between the results generated by these different predictors, Spearman correlation coefficients (SpR) were calculated between the predicted ΔΔ*G* values. The correlation between SAAMBE-3D and mCSM-PPI2 was found to be the highest (SpR = 0.584), while other pairs showed lower values (SAAMBE-3D and MutaBind2: SpR = 0.226, mCSM-PPI2 and MutaBind2: SpR = 0.352, Figure 3). These correlation measurements are important to show the degree of independence between the components of the ML ensemble. Ensembles containing excessively correlated methods would introduce unwanted biases into the predictions.

**Table 1:** Statistics of the Predictor Runs Collected for All Three Nanobodies and S-RBD Variants^a^

**statistics**	min	max	range	mean	StD	med	MAD	β	*p* (>0)
**dimension**	kcal × mol^–1^							dimensionless	
**mCSM-PPI2**	–2.93	2.88	5.81	0.39	0.70	0.35	0.34	0.23	77.5%
**SAAMBE-3D**	–1.64	4.35	5.99	0.76	0.66	0.68	0.33	0.32	91.7%
**MutaBind2**	–1.83	4.01	5.84	0.88	0.90	0.67	0.59	0.44	84.9%

a The dataset minima (min), maxima (max), ranges, means, standard deviations (StD), medians (med), median absolute deviations (MAD), Sarle’s bimodality coefficients (β), and greater-than-zero ratios (*p*(>0)) were calculated.

Another aspect we tested is how well the mutation types separate the data points into stabilizing and destabilizing mutations. Residues were grouped according to their chemical nature: (1) F, W, Y, H were considered aromatic, (2) R, H, K, D, E, S, T, N, Q were considered hydrophilic/nonpolar, (3) R, K were considered positively charged and (4) D, E were considered negatively charged. In addition, a group containing only G and a group containing only P were also considered. After selecting an appropriate group, the mutation types [*x* to *y*] and [*y* to *x*] were separated, where *x* is part of the selected group and *y* is not. From this point on, the nomenclature “out of group mutations” is used for the [*x* to *y*] cases and “into-group mutations” is used for the [*y* to *x*] cases. To assess the mutation type discriminative power of the predicted ΔΔ*G* values, ROC-AUC values were calculated based on the true and false out of group mutation rates at different ΔΔ*G* thresholds (Table 2 and SFigures 9–10). Kernel density estimates for the out of group and into-group mutations were also plotted for easy visual inspection of the ΔΔ*G* distributions (SFigures 11–12).

**Table 2:** Discriminative Ability of Different Predictors with Respect to the Out-of-Group Mutations (Positive Samples) and Into-Group Mutations (Negative Samples) Measured by Their ROC-AUC Values^a^

**predictor name/residue groups**	mCSM-PPI2	MutaBind2	SAAMBE-3D
**FWYH**	0.958	0.643	0.924
**RHKDESTNQ**	0.429	0.403	0.419
**RK**	0.635	0.576	0.582
**DE**	0.437	0.295	0.425
**G**	0.353	0.512	0.067
**P**	0.150	0.232	0.034

a Values far away from 0.5 (close to either 0 or 1) indicate a good discrimination ability. For example, in the case of SAAMBE-3D, the predicted ΔΔ*G* values can be easily separated if we consider only into proline and out of proline mutations (ROC-AUC = 0.034). In contrast, for MutaBind2 the predicted ΔΔ*G* values do not indicate whether a mutation is into glycine or out of glycine (ROC-AUC = 0.512).

Groups for which the mutation types achieve good separation are groups for which the AUC value is either close to 1 or 0, i.e., far away from 0.5. This analysis shows how these groups affect the ΔΔ*G* prediction result. Consensus results indicate that mutation of a nonaromatic residue to an aromatic one is generally predicted to be beneficial for the binding of the selected nanobodies to any S-RBD variant, while mutation of a nonproline residue to a proline disrupts the nanobody-antigen complex. Predictors can also vary widely in this regard; in the case of “into-glycine” mutations SAAMBE-3D shows a large separation (favoring out of glycine mutations as binding-promoting), while MutaBind2 shows no separation for this group. We would argue that these biases, especially the one concerning the aromatic residues, are important aspects of these ML methods during the nanobody design process.

Additional analysis regarding the agreement between the ML methods (SText9, STable2) and the effects of the Δ_1_ or Δ_2_ subvariant mutations (SText10, STable3, SFigure 13) can be found in the Supporting Information file.

**Figure 4:**
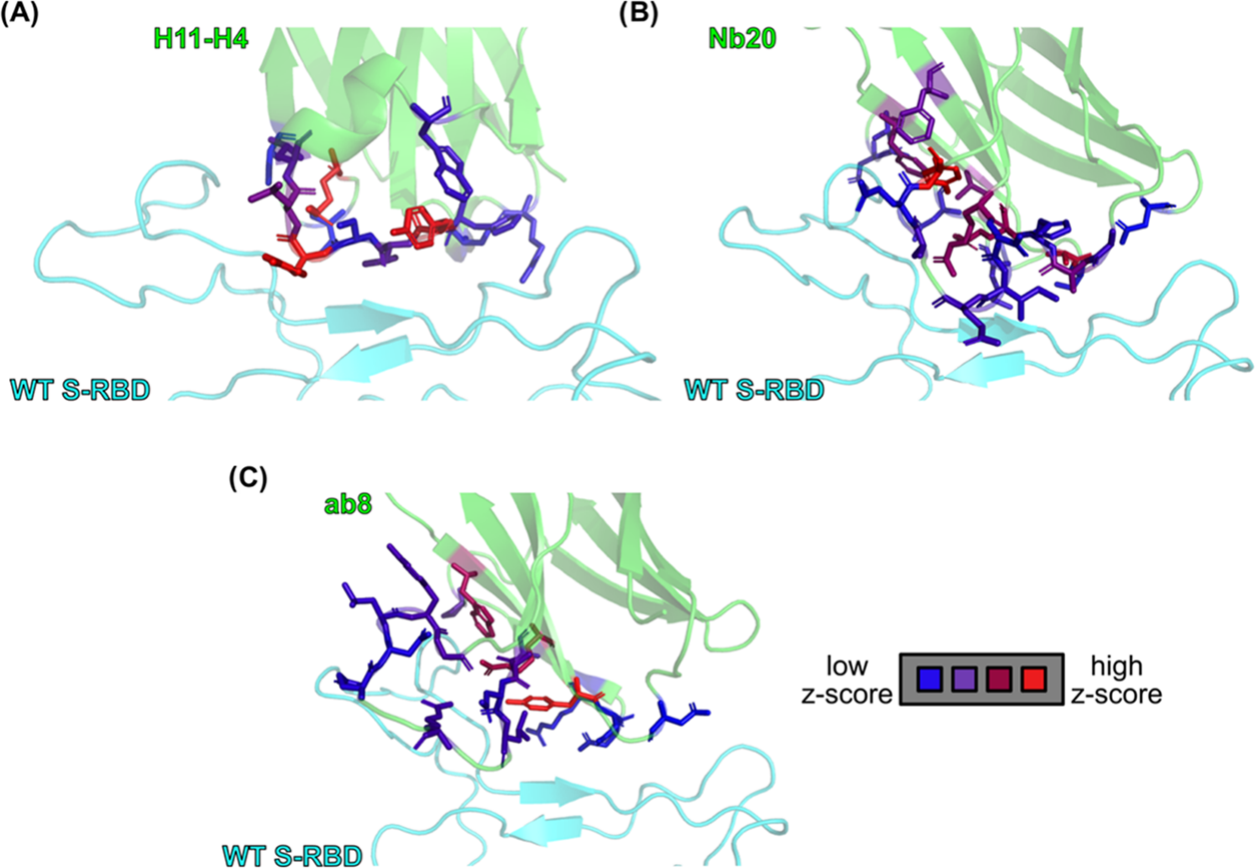
Interfacial residues colored according to their average absolute consensus z-scores on nanobodies H11-H4, Nb20, and ab8, shown in panels (A) (B), and (C), respectively. The color scale ranges from blue, indicating a low average absolute *z*-score, through purple, up to red, indicating a high average absolute *z*-score.

As a final analysis of the behavior of the predictor algorithms, we looked at the consensus z-scores of each mutation (see the Materials and Methods section for technical details) and ordered them increasingly (Table 3). Table 3 served as a template for selecting the appropriate nanobody mutations for MD analysis and subsequent bacterial expression and BLI measurements. To visualize the importance of the nanobody sites, we calculated the average absolute z-score for each site, i.e., the average of the absolute z-scores of the 19 different mutations for each specific interface amino acid. These averages were stored in pdb files and used to color the resulting structure (Figure 4). In the H11-H4 vs WT S-RBD complex the H11-H4 sites R52, Y101 and Y104 have the highest average absolute z-score, meaning that mutations at these sites are expected to have large effects on nanobody binding. R52 is in a salt bridge with the S-RBD site E484 and also forms a pi-cation stacking interaction with F490. It also stabilizes the position of the nanobody loop between H100-L106 through an H-bond with S103. Y101 and Y104 both participate in hydrophobic interactions, the former one with the S-RBD residue Y449, the latter one with L455, F456, Y489 and Q493. In Nb20 sites R31, A51 and Y104 are highlighted with the highest z-scores. R31 is, again, a key residue forming a salt bridge with E484 and a pi-cation interaction with F490, similar to R52 in H11-H4. A51 is part of a small hydrophobic core additionally formed by A29 and Y449. As seen in STable2, mutation of this alanine to larger hydrophobic residues is predicted to be favorable, expanding this hydrophobic interaction network. Interestingly, Y104 does not participate in any major interaction with the S-RBD, except for a possible H-bond with E484, but rather fills a cavity formed between the two proteins. Mutations of Y104 to smaller residues, such as E, T, P or A, result in unfavorable predicted ΔΔ*G* values. Finally, in nanobody ab8, sites W68, R71, and Y73 appear to be most sensitive to mutations. These residues are close to each other at the interface, in proximity with the critical S-RBD residue E484, presumably all responsible for its stabilization.

**Table 3:** Five Best Nanobody Mutations According to Their z-Scores^a^

	**WT S-RBD**			**S-RBD variant Δ**_**1**_			**S-RBD variant Δ**_**2**_		
**rank**	**H11-H4** (247)	**Nb20** (399)	**ab8** (342)	**H11-H4** (247)	**Nb20** (398)	**ab8** (342)	**H11-H4** (247)	**Nb20** (399)	**ab8** (342)
1	L106W	A51W	S79Y	L106W	A29F	S79Y	L106W	A29F	S79Y
2	L106Y	A29F	E127W	V102Y	A29Y	S79H	L106Y	A29Y	E127W
3	L106F	A29Y	L129Y	L106F	A51W	E127W	L106F	A29N	G131Y
4	V102Y	A51Y	L129F	L106Y	A48F	S79F	V102Y	A51W	L129Y
5	H100Y	A51H	L129W	L105Y	A29N	L129F	L105Y	R31Y	L129F

a In brackets is the total number of interfacial mutations considered, from which the 5 best were selected.

### MD Simulations, RING Analysis, and MM/GBSA Calculations

We selected 11 interesting H11-H4, Nb20 and ab8 mutants from Table 3 for MD simulations and subsequent MM/GBSA ΔΔ*H* calculations. In addition, simulations were performed for the H11-H4 double mutant V102Y, L106W in complex with the WT S-RBD and the mutant Y104D, which was predicted to be highly disruptive to complex formation. Analyses were performed for both WT S-RBD and Δ_1_ S-RBD complexes. Additionally, in order to select mutants for MM/GBSA analysis, we extracted additional information from the MD runs in the form of RMSD and clustering calculations. Table 4 summarizes the results of the MM/GBSA and clustering analysis runs. SFigure 14–19 show the RMSD time evolution of the complexes and the cluster centers as protein ensembles. Since the *K*_d_ value of the WT Nb20 is very low, suggesting a highly optimized interface with little room for improvement, we focused our efforts on the H11-H4 and ab8 nanobodies, where the high computational demand of MD would be a worthwhile investment.

**Table 4:**
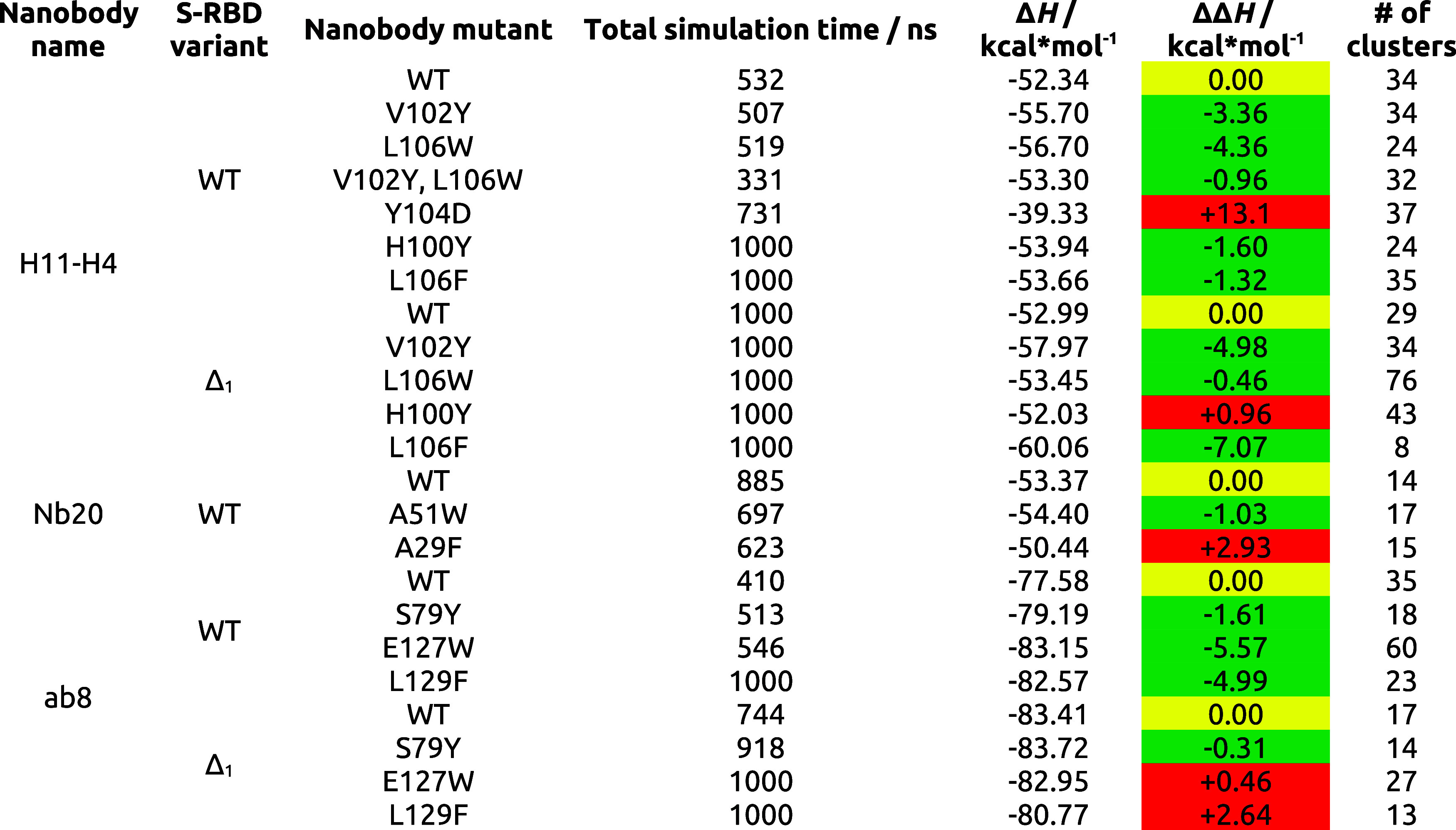
Numerical Data Obtained from the MD Simulations for the Different Nanobody Mutants^a^

a Red ΔΔ*H* cells indicate worse binding, while green ΔΔ*H* cells indicate better binding compared to the WT nanobody complex (yellow cells).

To determine the qualitative cause of the quantitative binding differences, we subjected the best binding mutant and the WT nanobody complexes to RING analysis.^35,36^ Comparing the WT H11-H4/WT S-RBD complex with the L106W mutant H11-H4/WT S-RBD complex, the resulting ΔΔ*H* is −4.36 kcal/mol. Changes in the average count of interactions per frame are from 10.7 to 11.9 for van der Waals (VdW) interactions, from 8.0 to 9.1 for H-bonds, and from 4.1 to 4.8 for pi-pi stacking interactions. Interface residue–residue interaction networks can be seen on SFigures 20 and 21, which show high similarity. We examined the behavior of the residues at this mutation site. L106 and W106 both interact with E484, although these interactions are transient and backbone-mediated H-bonds, mostly independent of the side chains. W106 forms an additional VdW interaction with G485. Inspection of the cluster center structures shows that the introduction of the bulky tryptophan facilitates an intrachain edge-to-face interaction with Y59, a residue which does not interact strongly with the L106 at the WT site.

When the Δ_1_ S-RBD protein is considered in complex with H11-H4, the nanobody mutation L106F results in the largest increase in binding affinity, with a ΔΔ*H* of −7.07 kcal/mol. In fact, this is the largest negative ΔΔ*H* of all the complexes studied. This large change is also reflected in the average number of interactions: the average number of VdW interactions per frame increases from 11.4 to 12.5, for H-bonds this change is from 9.5 to 10.4, pi–pi stacking interactions increase in number from 3.8 to 4.7, and salt bridges increase from 1.2 to 1.4, when the L106F mutation is introduced. Site 106 has little or no direct interaction with the S-RBD. In case of the WT complex, L106 forms a weak H-bond through its backbone with E484 (see SFigures 22 and 23). In the mutant complex F106 forms hydrophobic interactions with the aliphatic side of the E484 glutamic acid. This is due to the conformational constraint of F106 imposed by the presence of Y59 on the same chain.

Switching from the WT Nb20/WT S-RBD complex to the A51W Nb20/WT S-RBD complex results in a calculated ΔΔ*H* of −1.03 kcal/mol. As expected, such a small improvement in binding affinity is not reflected in the average interaction counts of the different secondary chemical bonds: VdW counts are 14.1 and 14.8, H-bond counts change from 8.5 to 8.3, and salt bridge counts are 0.5 and 0.7 for the WT nanobody and A51W mutant nanobody complexes, respectively. In this case, however, the introduction of W51 can be easily spotted on the interaction network diagram (SFigures 24 and 25). Instead of a single A51-Y449 interaction, W51 forms interactions with Y449, L452, Q493 and S494. This means that while A51 forms an average of 0.53 interactions per frame, W51, due to its size, is able to form 2.33 interactions. Although most of these interactions are VdW contacts, the tryptophan is large enough to reach the backbone oxygen of Y449 and form an interfacial H-bond with its Hε1 hydrogen atom.

In the case of the ab8/WT S-RBD complex, the E127W proved to be the best nanobody mutation with the lowest calculated ΔΔ*H* of −5.57 kcal/mol. Interestingly, in this case, the average number of the different interaction types is not significantly different between the two complexes. The average number of VdW interactions increases from 16.0 to 17.5, for the H-bonds this negligible change is from 14.8 to 14.9, while for pi-pi stacking interactions this number decreases from 1.9 to 1.7. In addition, the two interaction networks seen on SFigures 26 and 27 are also very similar. Neither L106, nor W106 appear in these networks. Looking at the first few cluster centers, it can be seen that L106 and W106 both face the solvent bulk, pointing away from the S-RBD. Thus, according to the simulations, the effect of this mutation acts indirectly on the nanobody/S-RBD interface, via allosteric or solvation effects.

For the ab8/Δ_1_ S-RBD complex, the S79Y mutation was the most favorable. The corresponding ΔΔ*H* value is −0.31 kcal/mol, much lower than for the proposed changes for the ab8/WT S-RBD complex. The S79Y mutation causes an increase in the average number of VdW interactions from 17.4 to 18.2, a decrease in H-bonds from 15.4 to 14.4, and a slight increase in the average number of pi-pi stacking interactions from 1.7 to 1.8. Similar to several previous cases, the interaction profile of the WT and S79Y nanobody Δ_1_ S-RBD complexes is almost identical (SFigures 28 and 29). However, in contrast to that seen in case of the ab8/WT S-RBD complex, S79 forms a strong H-bond with the critical S-RBD residue E484, which is lost when Y79 is introduced, forming only relatively weak van der Waals contacts with E484. Although homochain interactions are not indicated on the networks, in the cluster center structures Y79 is close to the hydrophobic nanobody residues W68 and P82, suggesting intrachain interactions as the rationale for this type of mutation choice.

### Production and Purification of Mutant Nanobodies

We aimed to compare the *in vitro* binding strength of the *in silico* proposed mutant nanobody versions to the WT and Δ_1_ S-RBD variants with the binding strength of the previously tested WT nanobodies.^15^ After expression, the size and purity of the nanobodies were validated by SDS-PAGE (SFigures 7–8). The expression yields are summarized in STable1.

The production of two of the mutants (ab8 S79Y and ab8 L129F, see STable1) proved unsuccessful even after several attempts. In these cases, while Ni-IMAC purification of the target protein fused to ecotin was still successful (SFigures 8 and 6), the target protein precipitated during cleavage of the fusion construct, indicating that the nanobody itself is unstable in solution. The ML based techniques used here mostly favored the mutations from small to medium sized residues (e.g., S, E, L, H, etc.) to larger aromatic residues (Y, W, F). It has been previously reported that aromatic residues are indeed more abundant at protein–protein interfaces.^37^ This can be attributed to the tendency of large hydrophobic residues (such as those residues with aromatic side chains) to bury themselves and displace water from the interface. Although this mechanism favors the formation of interfaces, it also destabilizes proteins in their monomeric form. In addition to reducing the solubility of the protein, these changes can lead to monomer misfolding or even the formation of homomultimeric aggregates. We hypothesize that the failure to purify the ab8 mutants S79Y and L129F is due to these reasons.

All other mutants were successfully produced and purified. (SFigures 7 and 1–5) We can observe that the production yield of the H11-H4 nanobody is low compared to the yield of the ab8 mutant. This is a characteristic of the H11-H4 nanobody, as we have already observed with the WT nanobodies as well.^15^ However, the amount of material required for further protein–protein binding assays was successfully obtained.

### Biolayer Interferometry Measurements of the Mutant Nanobodies

After the expression of the selected mutant variants, we performed a BLI binding assay between the nanobody mutants and both the wild-type and the Δ_1_ variant S-RBD. First, the complex formation of the WT H11-H4 nanobody and its four mutants (V102Y, L106W, H100Y, L106F) with the two S-RBD variants was measured.

**Figure 5:**
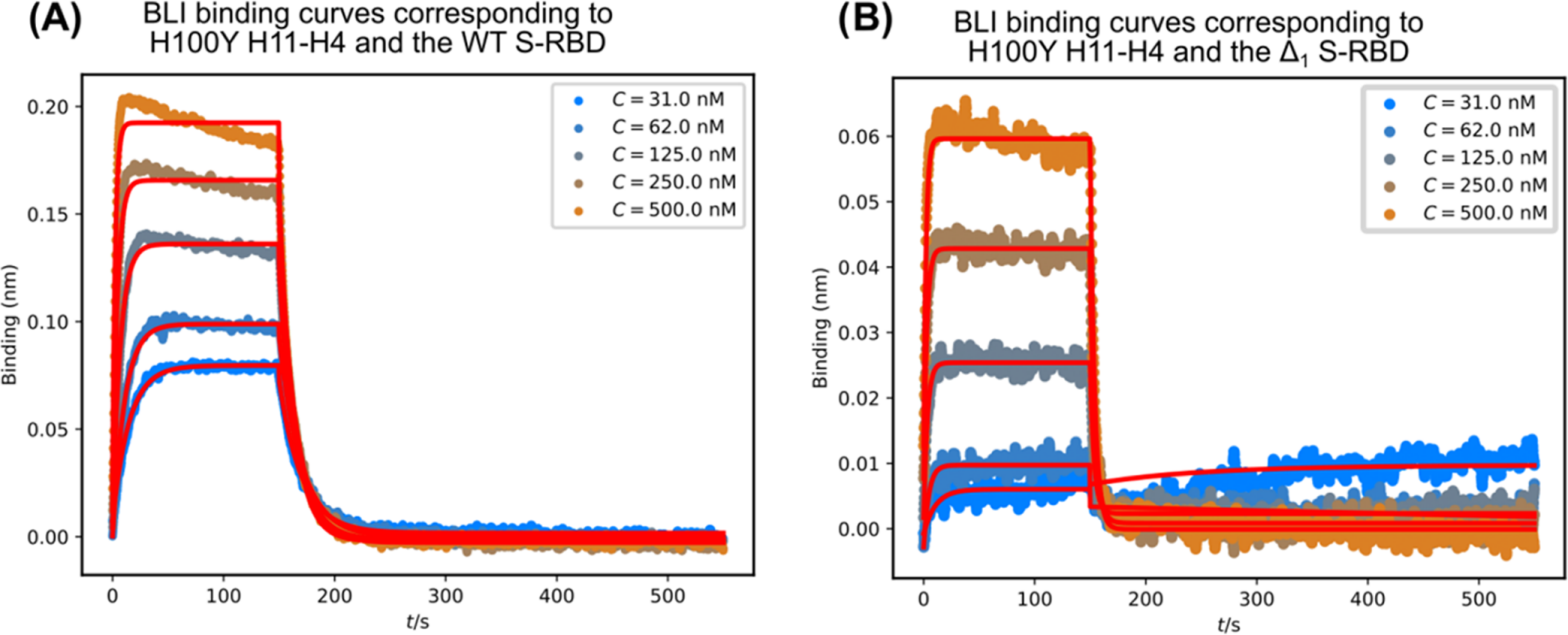
Binding of the H11-H4 H100Y mutant nanobody to (A) Spike protein S-RBD wild-type variant and (B) S-RBD Δ_1_ variant at different concentrations. The measurements are characterized by fast association and fast dissociation kinetics relative to the WT H11-H4 nanobody.

Analyzing the nature and kinetics of the binding and comparing them with their WT counterparts, all the mutants show faster association to the S-RBD, as well as faster dissociation (Figure 5A and SFigure 30–32). This means that the binding partners can easily find each other, leading to the hypothesis that the nanobody binding interface has become more available to the S-RBD. However, the fast release of the nanobody from the complex indicates that the resulting interface is weaker, suggesting suboptimal residue–residue interactions. Comparing the *K*_d_ constants in the summary, it can be seen that none of these mutants can produce better binding values than that of the WT (Table 5).

**Table 5:**
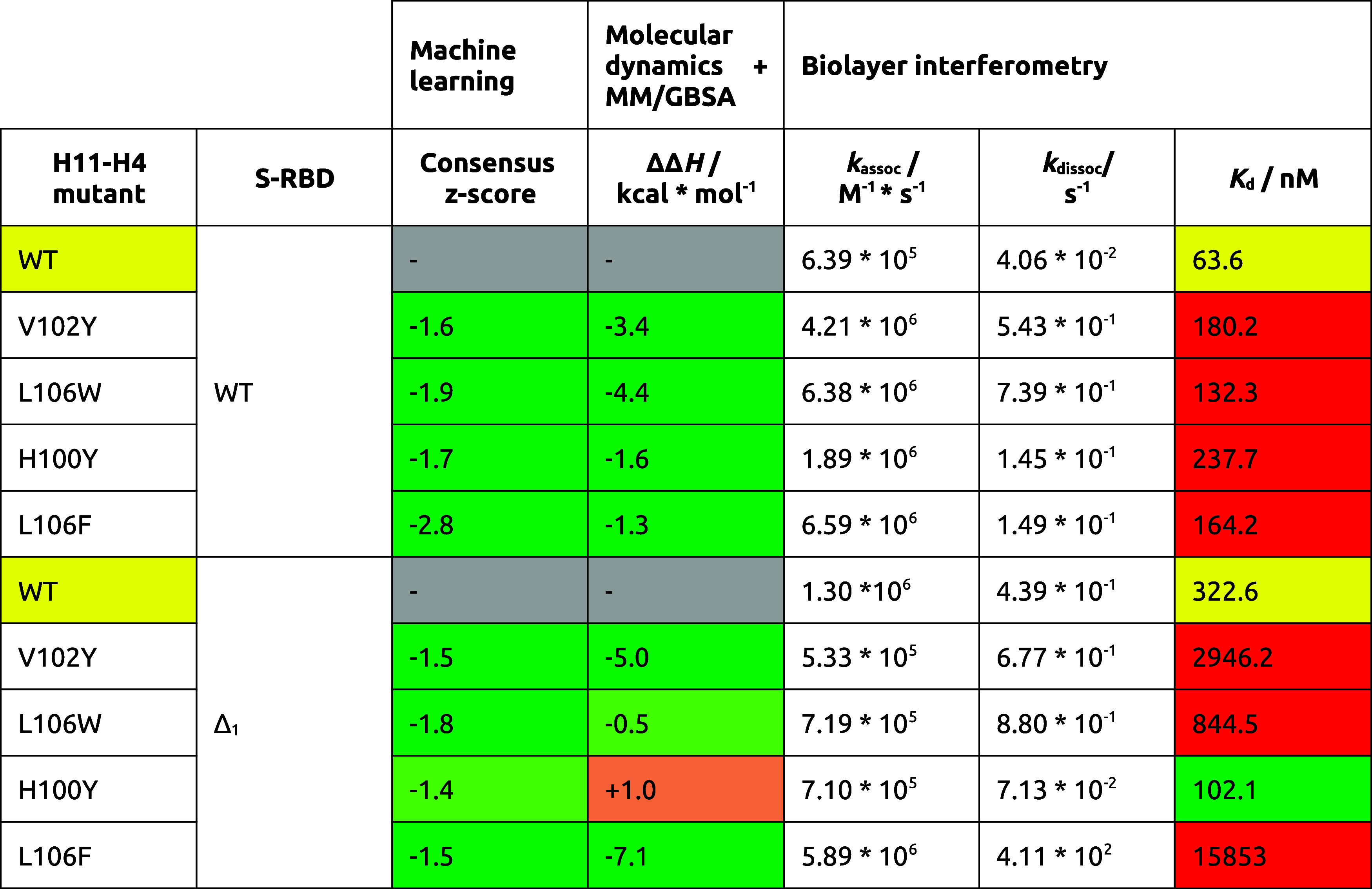
Collected Values Summarizing the Machine Learning and the BLI Measurements for the WT H11-H4 and Its Mutants Studied with Both S-RBD Variants

These mutant nanobodies were also tested with the Δ_1_ S-RBD variant. However, the resulting binding was very weak in most of the cases (Figure 5B and in SFigures 30–32/B). For two of the mutants, namely the L106F and H100Y, the dissociation values were better than in the case of the wild-type nanobody, but only in the case of H100Y was it sufficient to obtain a better *K*_d_ value and a better binding result.

H11-H4 showed strong binding to both the wild-type and Δ_1_ S-RBD protein, making it difficult to design better mutants. As expected, the binding to the WT S-RBD was not improved, while the Δ_1_ S-RBD mutants mostly showed worse binding. This may be due to the bias of the predictors toward aromatic substitutions that we noted earlier. At certain interface sites, the introduction of bulky lipophilic residues may help complex formation, but steric clashes may also disrupt these already well designed interfaces, increasing the *K*_d_ value.

**Figure 6:**
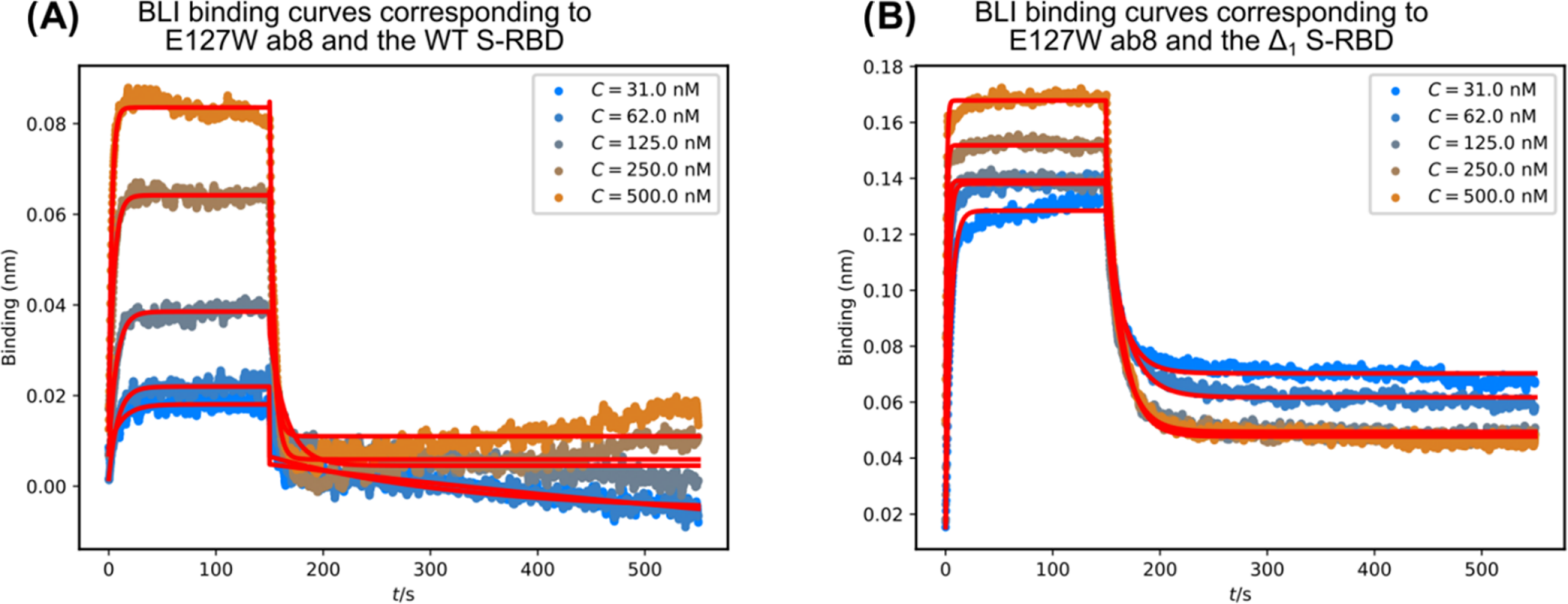
Binding of the ab8 E127W mutant nanobody to (A) WT S-RBD and (B) Δ_1_ S-RBD variant at different concentrations. The measurements are characterized by fast association and fast dissociation kinetics.

In the case of the E127W mutant ab8 nanobody, regardless of which S-RBD variant is the partner, the binding curves are well behaved and show similar binding kinetics. Both association and dissociation are fast. At the same concentrations, the binding curve is similar to that of the WT ab8/WT S-RBD, but in the case of the Δ_1_ S-RBD, the binding curve shows much stronger binding due to extremely fast association (Figure 6). Numerical data for these kinetic analyses are shown in Table 6.

**Table 6:**
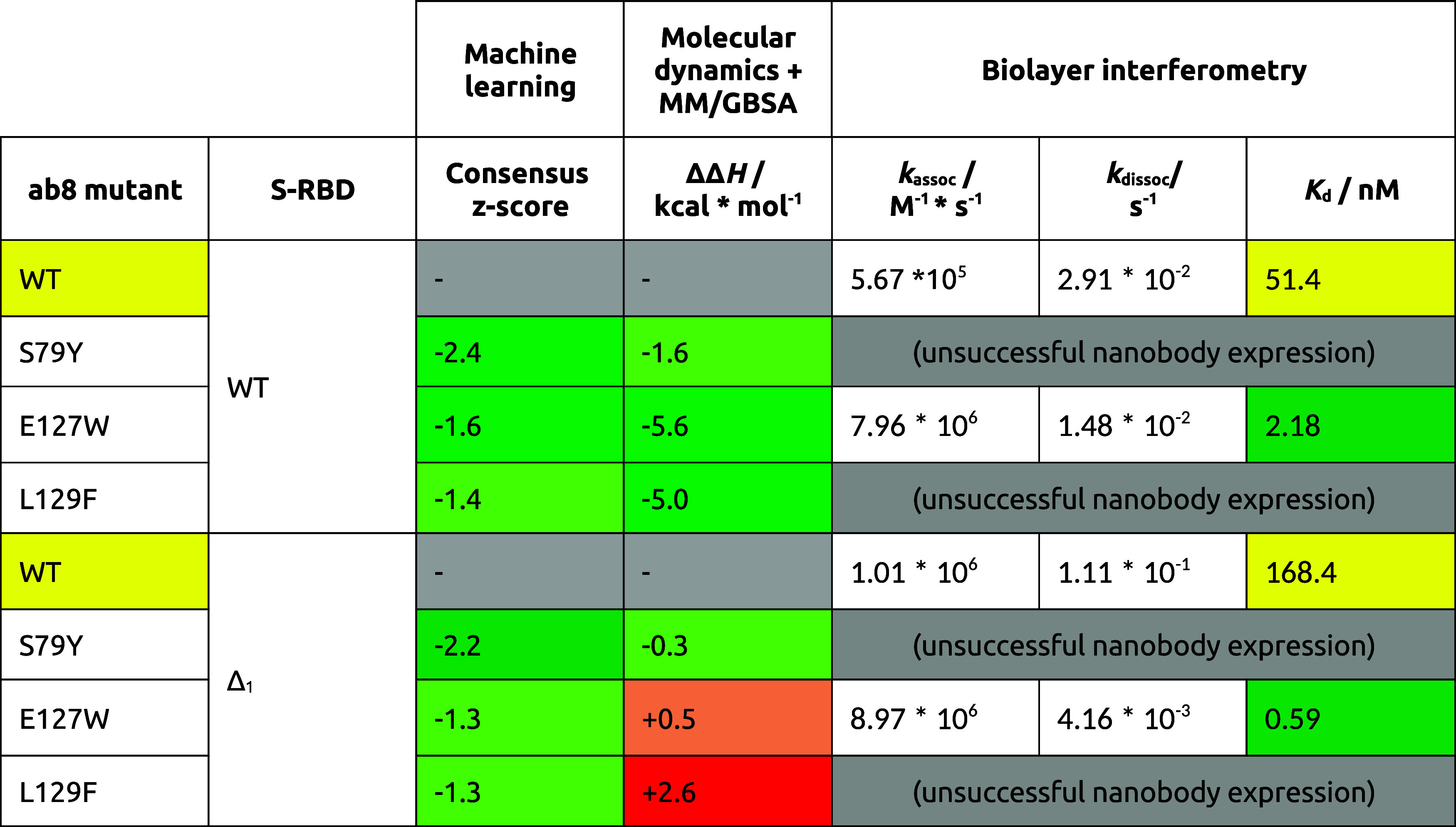
Collected Values Summarizing the Machine Learning and the BLI Measurements for the WT ab8 and Its Mutants Studied with Both S-RBD Variants

## Conclusions

The rational design of antibody mimetics is a nontrivial task, meaning that direct exploration of the mutation space by *in vitro* experimental means is usually very expensive and time-consuming. This design pattern can be complemented by *in silico* studies, which promise to narrow down the mutation options to be tested, lowering research costs and accelerating the development of potent binders. Here, we proposed and analyzed a possible *in silico* coupled *in vitro* nanobody design workflow against two SARS-CoV-2 S-RBD variants.

By testing our proposed nanobody design workflow, we were able to identify the strengths and weaknesses of the current state of *in silico* assisted nanobody design. Our three-step pipeline consisted of: (1) ML-assisted selection of nanobody mutants against two SARS-CoV-2 S-RBD variants, (2) MD- and MM/GBSA-assisted prefiltering of ML-suggested mutants, and (3) expression of nanobody mutants and their BLI measurements as an experimental filtering and validation method. We considered three different nanobodies, namely H11-H4, Nb20, and ab8, as well as two different variants of the S-RBD. We analyzed the behavior of three ML methods: mCSM-PPI2, MutaBind2 and SAAMBE-3D. We showed how their predictions are distributed, how these methods correlate with each other, as well as how these methods prefer different mutation types, such as the mutation of a nonaromatic residue to an aromatic one. The consensus z-scores of the mutations were used to map the importance of the mutations at the nanobody/S-RBD interface. The interactions of the most important sites were also analyzed.

We further analyzed the nanobody mutants proposed by ML using MD and MM/GBSA. It is evident from the collected data that the order determined by the consensus z-score is not necessarily in agreement with the order determined by the ΔΔ*H* values from the MM/GBSA runs. One possible explanation for this is that our MM/GBSA runs ignored the entropic effects. (The calculations either failed or the variance of the ΔΔ*G* values became unacceptable when we tried to include the entropic contribution using the proposed techniques (quasi-harmonic approximation, interaction entropy, or C2 entropy^38^) (data not shown)). However, a much more likely explanation for the discrepancy is the use of single-trajectory MM/GBSA approximations (which was unfortunately necessary to achieve satisfactory accuracy), the low sampling rate of the full conformational space (even after 1 μs of simulation), and the low accuracy of ML methods in the case of these protein complexes.

Finally, we also identified an important weakness in the optimization process: ML methods do not optimize for protein stability, but only for successful binding. This may explain the failure to express two ab8 mutants (S79Y and L129F), as their separation and purification from the fusion partner ecotin remained unresolved. Nevertheless, we successfully synthesized five different nanobody mutants and showed that H11-H4 H100Y indeed binds to the Δ_1_ S-RBD more strongly than its WT counterpart, and that E127W ab8 binds to both S-RBD variants with higher affinity than the WT.

Further refinements to the proposed workflow could potentially lead to more accurate *in silico* affinity predictions. Including more than three ML methods based on several diverse techniques would result in more trustworthy ensemble predictions. Since these methods work on the WT protein structure, a short conformational search prior to the ML runs could provide several different input conformations, yielding a distribution of ΔΔ*G* predictions rather than a single value. This could also be achieved with a direct feedback loop between the ML runs and the MD runs. We also pointed out the lack of stability prediction steps in the workflow, which turned out to be an important missing filter. Finally, more accurate ΔΔ*H* or ΔΔ*G* predictions could also be extracted from the MD runs, either by including the entropy calculations into the MM/GBSA protocol, or by using alchemical methods (e.g., thermodynamic integration, free energy perturbation, or umbrella sampling).

## Data Availability

Inputs and outputs for the mCSM-PPI2, MutaBind2, and SAAMBE-3D ML Web servers, along with scripts processing these, as well as pdb formatted structure files used in this study are provided on GitHub with the following link: https://github.com/fazekaszs/nanobodyProject (doi.: 10.5281/zenodo.12073361).

## Supporting Information Available

SText1–8: DNA and protein sequences of the nanobodies and S-RBD variants, SFigures 1–6: size exclusion chromatograms of the expressed proteins, SFigures 7–8: SDS-PAGE of the protein purification steps, STable1: expression yields, SFigures 9–12: predicted ΔΔ*G* separation ability of the different residue/mutation groupings, SText9+STable2: complex-wise and predictor-wise analysis of mutations, SText10+STable3+SFigure 13: ΔΔΔ*G* analysis of the nanobody mutations and S-RBD variants, SFigures 14–19: RMSD curve of the MD trajectories and nanobody complex clustering structural ensembles, SFigures 20–29: RING (residue interaction graph) analysis of the complexes based on the MD runs, SFigures 30–32: BLI nanobody/S-RBD affinity measurement curves (PDF)

## Abbreviations

ACE2: angiotensin converting enzyme 2
AMP: ampicillin
BLI: biolayer interferometry
COVID-19: coronavirus disease (emerged in 2019)
CHO: Chinese Hamster Ovary
CV: column volume
E. coli: *Escherichia coli*
IDT: integrated DNA Technologies
IMAC: immobilized metal-ion affinity chromatography
IPTG: isopropyl-β-thiogalactoside
ITC: isothermal titration calorimetry
LB: Luria broth
LIE: linear interaction energy
LRA: linear response approximation
MAD: median absolute deviation
MD: molecular dynamics
ML: machine learning
MM/GBSA: molecular mechanics/generalized Born and surface area solvation
MM/PBSA: molecular mechanics/Poisson–Boltzmann and surface area solvation
NMR: nuclear magnetic resonance spectroscopy
OD_600_: optical density measured at 600 nm
PBS: phosphate buffer saline
RMSD: root mean squared deviation
ROC-AUC: receiver operating characteristic curve–area under the curve
SARS-CoV-2: severe acute respiratory syndrome coronavirus 2
SDS-PAGE: sodium dodecyl sulfate polyacrylamide gel electrophoresis
SEC: size exclusion chromatography
SpR: Spearman’s correlation coefficient
SPR: surface plasmon resonance
S-RBD: spike protein receptor binding domain
SSM: site saturation mutagenesis
StD: standard deviation
VdW: van der Waals interaction
WT: wild type
