# Supplementary material for: Evaluation of an Affinity-Enhanced Anti-SARS-CoV2 Nanobody Design Workflow Using Machine Learning and Molecular Dynamics
Source: J Chem Inf Model. 2024 Oct 2;64(19):7626–38. doi: 10.1021/acs.jcim.4c01023 (PMC11481066; doi:10.1021/acs.jcim.4c01023)
Supplement: Supplementary file 1 — ci4c01023_si_001.pdf [file ci4c01023_si_001.pdf]

Supplementary information

Evaluation of an Affinity-Enhanced anti-SARS-CoV2 Nanobody Design Workflow Using Machine Learning and Molecular Dynamics

Zsolt Fazekas[3,4]**, Dóra Nagy-Fazekas[3,4]**, Boglárka Mária Shilling-Tóth[5], Péter Ecsédi[5], Pál Stráner[2,4], László Nyitray[5] and András Perczel[1,2,4,] *

[1]Medicinal Chemistry Research Group, HUN-REN Research Centre for Natural Sciences, Magyar Tudósok Körútja 2, H-1117 Budapest, Hungary

[2]HUN-REN-ELTE Protein Modeling Research Group, Hungarian Research Network (HUN-REN), Institute of Chemistry, Eötvös Loránd University, Pázmány Péter sétány 1/A, Budapest, H-1117, Hungary

[3]Hevesy György PhD School of Chemistry, Institute of Chemistry, Eötvös Loránd University, Budapest, Pázmány Péter sétány. 1/A, Budapest, H-1117, Hungary

[4]Laboratory of Structural Chemistry and Biology, Institute of Chemistry, Eötvös Loránd University, Pázmány Péter sétány 1/A, Budapest, H-1117, Hungary

[5]Eötvös Loránd University, Department of Biochemistry, Pázmány Péter sétány 1/C, Budapest, H-1117, Hungary

* Corresponding author
** These authors contributed equally to this article. (shared first authors)

- perczel.andras@ttk.elte.hu, perczel.andras@ttk.hu

SText1 DNA sequence and data of the expressed wild type spike protein RBD:

ATGTTCGTGTTTCTGGTGCTGCTGCCTCTGGTGTCCAGCCAGCGGGTGCAGCCCACCGA
ATCCATCGTGCGGTTCCCCAATATCACCAATCTGTGCCCCTTCGGCGAGGTGTTCAATGC
CACCAGATTCGCCTCTGTGTACGCCTGGAACCGGAAGCGGATCAGCAATTGCGTGGCCG
ACTACTCCGTGCTGTACAACTCCGCCAGCTTCAGCACCTTCAAGTGCTACGGCGTGTCCC
CTACCAAGCTGAACGACCTGTGCTTCACAAACGTGTACGCCGACAGCTTCGTGATCCGG
GGAGATGAAGTGCGGCAGATTGCCCCTGGACAGACAGGCAAGATCGCCGACTACAACT
ACAAGCTGCCCGACGACTTCACCGGCTGTGTGATTGCCTGGAACAGCAACAACCTGGAC
TCCAAAGTCGGCGGCAACTACAATTACCTGTACCGGCTGTTCCGGAAGTCCAATCTGAA
GCCCTTCGAGCGGGACATCTCCACCGAGATCTATCAGGCCGGCAGCACCCCTTGTAACG
GCGTGGAAGGCTTCAACTGCTACTTCCCACTGCAGTCCTACGGCTTTCAGCCCACAAAT
GGCGTGGGCTATCAGCCCTACAGAGTGGTGGTGCTGAGCTTCGAACTGCTGCATGCCCC
TGCCACAGTGTGCGGCCCTAAGAAAAGCACCAATCTCGTGAAGAACAAATGCGTGAAC
TTCCACCATCACCATCACCATTGATAA

SText2 DNA sequence and data of the expressed delta variant spike protein RBD:

ATG TTT GTA TTT CTC GTG CTC CTC CCT CTC GTT TCC AGT CAA AGG GTG CAA
CCA ACT GAA AGC ATT GTC CGA TTT CCC AAC ATC ACA AAT CTC TGT CCT TTT
GGG GAG GTA TTC AAT GCT ACC AGA TTC GCC TCT GTT TAT GCT TGG AAT CGG
AAA CGT ATC AGT AAC TGC GTG GCT GAC TAC TCT GTA TTG TAC AAT TCA GCA
TCA TTC TCC ACA TTC AAA TGC TAC GGG GTG AGT CCC ACC AAA CTT AAT GAT
CTC TGT TTC ACC AAC GTA TAT GCA GAC TCC TTC GTT ATT CGG GGA GAT GAG
GTT AGA CAG ATT GCA CCT GGG CAA ACT GGC AAG ATC GCA GAT TAC AAT TAT
AAA CTG CCT GAT GAT TTC ACA GGT TGT GTG ATC GCT TGG AAT TCC AAC AAC
CTG GAT AGC AAG GTT GGA GGG AAT TAT AAT TAC AGG TAT CGA CTT TTT CGC
AAA TCC AAC CTC AAA CCA TTT GAA CGT GAC ATT TCA ACT GAG ATC TAC CAA
GCT GGG AGC AAA CCA TGT AAT GGC GTG GAG GGA TTC AAC TGC TAC TTT CCT
CTC CAG TCA TAC GGC TTT CAA CCT ACT AAC GGA GTT GGT TAT CAG CCT TAT
AGA GTG GTG GTG TTG AGT TTT GAG CTT CTC CAT GCC CCT GCA ACT GTG TGC
GGG CCC AAG AAA AGC ACC AAT CTT GTG AAG AAC AAA TGC GTA AAC TTT CAC
CAC CAC CAT CAC CAT TAG

SText3 DNA sequence of the ecotin fusion protein sequence (NdeI and XhoI restriction sites are highlighted)

CAT ATG AAG ACG ATC TTA CCT GCT GTG CTT TTC GCT GCT TTT GCA ACG ACT AGT
GCC TGG GCC GCA GAA TCT GTT CAG CCT CTG GAA AAA ATC GCG CCT TAT CCC CA
G GCT GAA AAG GGC ATG AAA CGC CAA GTC ATC CAA CTT ACC CCG CAA GAG GAT
GAA TCG ACC TTG AAA GTG GAA CTG TTA ATC GGG CAG ACA TTA GAG GTT GAC TG
C AAC CTT CAT CGT CTT GGC GGC AAA TTA GAG AAC AAG ACC TTG GAG GGA TGG
GGG TAC GAC TAC TAT GTG TTT GAT AAG GTC AGT TCT CCA GTC TCG ACT ATG AT
G GCT TGC CCT GAT GGG AAA AAG GAG AAG AAG TTT GTT ACC GCC TAT TTA GGC
GAC GCT GGA ATG CTT CGC TAC AAC AGT AAG TTA CCC ATC GTG GTT TAT ACG CC
C GAT AAT GTT GAC GTT AAA TAC CGC GTT TGG AAG GCA GAA GAG AAA ATC GAC

AAT GCG GTT GTG CGC GGA TCA ACT AGT GGT TCT GGT CAT CAC CAT CAC CAT CA
C TCC GCG GGT CTG GTG CCA CGC GGA TCC GAA TTC GAG CTC CGT CGA CAA GCT T
GC GGC CGC CTC GAG

SText4 DNA sequence of the expressed nanobodies

ab8:

GAA GTT CAG CTG GTT GAA TCT GGT GGT GGT CTG GTT CAG CCG GGT GGT TCT CT
G CGT CTG TCT TGC GCG GCG TCT GGT TTC ACC TTC GAC GAC TAC GCG ATG TCT T
GG GTT CGT CAG GCG CCG GGT AAA GGT CTG GAA TGG ATC GGT CGT ATG TAC AA
C AAC GGT CGT ACC TCT TAC AAC CCG TCT CTG AAA TCT CTG GTT ACC ATC TCT C
GT GAC AAC TCT AAA AAC ACC CTG TAC CTG CAG ATG AAC TCT CTG CGT GCG GAA
GAC ACC GCG ACC TAC TAC TGC GCG CGT GAC AAC CTG GGT TAC CGT CCG TCT GA
A AAC CTG TAC GGT ATG GAC GTT TGG GGT CAG GGT ACC ACC GTT ACC GTT TCT T
CT

ab8 E127W:

GAA GTT CAG CTG GTT GAA TCT GGT GGT GGT CTG GTT CAG CCG GGT GGT TCT CT
G CGT CTG TCT TGC GCG GCG TCT GGT TTC ACC TTC GAC GAC TAC GCG ATG TCT T
GG GTT CGT CAG GCG CCG GGT AAA GGT CTG GAA TGG ATC GGT CGT ATG TAC AA
C AAC GGT CGT ACC TCTTAC AAC CCG TCT CTG AAA TCT CTG GTT ACC ATC TCT CG
T GAC AAC TCT AAA AAC ACC CTGTAC CTG CAG ATG AAC TCT CTG CGT GCG GAA G
AC ACC GCG ACC TAC TAC TGC GCG CGT GAC AAC CTG GGT TAC CGT CCG TCT TGG
AAC CTG TAC GGT ATG GAC GTT TGG GGT CAG GGT ACC ACC GTT ACC GTT TCT TC
T

ab8 S79Y:

GGA TCC GAA GTT CAG CTG GTT GAA TCT GGT GGT GGT CTG GTT CAG CCG GGT GG
T TCT CTG CGT CTG TCT TGC GCG GCG TCT GGT TTC ACC TTC GAC GAC TAC GCG AT
G TCT TGG GTT CGT CAG GCG CCG GGT AAA GGT CTG GAA TGG ATC GGT CGT ATG
TAC AAC AAC GGT CGT ACC TAC TAC AAC CCG TCT CTG AAA TCT CTG GTT ACC AT
C TCT CGT GAC AAC TCT AAA AAC ACC CTG TAC CTG CAG ATG AAC TCT CTG CGT G
CG GAA GAC ACC GCG ACC TAC TAC TGC GCG CGT GAC AAC CTG GGT TAC CGT CC
G TCT GAA AAC CTG TAC GGT ATG GAC GTT TGG GGT CAG GGT ACC ACC GTT ACC
GTT TCT TCT

ab8 L129F:

GGA TCC GAA GTT CAG CTG GTT GAA TCT GGT GGT GGT CTG GTT CAG CCG GGT GG
T TCT CTG CGT CTG TCT TGC GCG GCG TCT GGT TTC ACC TTC GAC GAC TAC GCG AT
G TCT TGG GTT CGT CAG GCG CCG GGT AAA GGT CTG GAA TGG ATC GGT CGT ATG
TAC AAC AAC GGT CGT ACC TCT TAC AAC CCG TCT CTG AAA TCT CTG GTT ACC ATC
TCT CGT GAC AAC TCT AAA AAC ACC CTG TAC CTG CAG ATG AAC TCT CTG CGT GC
G GAA GAC ACC GCG ACC TAC TAC TGC GCG CGT GAC AAC CTG GGT TAC CGT CCG
TCT GAA AAC TTC TAC GGT ATG GAC GTT TGG GGT CAG GGT ACC ACC GTT ACC GT
T TCT TCT

H11-H4:

CAG GTT CAG CTG GTT GAA TCT GGT GGT GGT CTG ATG CAG GCG GGT GGT TCT CT G CGT CTG TCT TGC GCG GTT TCT GGT CGT ACC TTC TCT ACC GCG GCG ATG GGT T GG TTC CGT CAG GCG CCG GGT AAA GAA CGT GAA TTC GTT GCG GCG ATC CGT TGG TCT GGT GGT TCT GCG TAC TAC GCG GAC TCT GTT AAA GGT CGT TTC ACC ATC TCT CGT GAC AAA GCG AAA AAC ACC GTT TAC CTG CAG ATG AAC TCT CTG AAA TAC G AA GAC ACC GCG GTT TAC TAC TGC GCG CAG ACC CAC TAC GTT TCT TAC CTG CTG TCT GAC TAC GCG ACC TGG CCG TAC GAC TAC TGG GGT CAG GGT ACC CAG GTT AC C GTT TCT TCT

H11-H4 H100Y:

CAG GTG CAA CTG GTC GAG TCT GGG GGA GGA TTG ATG CAG GCT GGG GGC TCT C TG AGA CTC TCC TGT GCA GTC TCT GGA CGC ACC TTC AGT ACC GCT GCG ATG GGC TGG TTC CGC CAG GCT CCA GGG AAG GAG CGT GAG TTT GTA GCA GCT ATT AGG TG G AGT GGT GGT AGC GCA TAC TAT GCA GAC TCC GTG AAG GGC CGA TTC AAC ATC TCC AGA GAC AAG GCC AAG AAC ACG GTA TAT CTG CAA ATG AAC AGC CTG AAA T AT GAG GAC ACG GCC GTT TAT TAC TGT GCA CAA ACG TAT TAT GTT TCT TAT CTC CTT AGC GAC TAT GCC ACT TGG CCT TAT GAC TAC TGG GGC CAG GGG ACC CAG GT C ACC GTC TCC TCC TAA

H11-H4 V102Y:

CAG GTT CAG CTG GTT GAA TCT GGT GGT GGT CTG ATG CAG GCG GGT GGT TCT CT G CGT CTG TCT TGC GCG GTT TCT GGT CGT ACC TTC TCT ACC GCG GCG ATG GGT T GG TTC CGT CAG GCG CCG GGT AAA GAA CGT GAA TTC GTT GCG GCG ATC CGT TGG TCT GGT GGT TCT GCG TAC TAC GCG GAC TCT GTT AAA GGT CGT TTC ACC ATC TCT CGT GAC AAA GCG AAA AAC ACC GTT TAC CTG CAG ATG AAC TCT CTG AAA TAC G AA GAC ACC GCG GTT TAC TAC TGC GCG CAG ACC CAC TAC TAC TCT TAC CTG CTG TCT GAC TAC GCG ACC TGG CCG TAC GAC TAC TGG GGT CAG GGT ACC CAG GTT AC C GTT TCT TCT

H11-H4 L106F:

CAG GTG CAA CTG GTC GAG TCT GGG GGA GGA TTG ATG CAG GCT GGG GGC TCT C TG AGA CTC TCC TGT GCA GTC TCT GGA CGC ACC TTC AGT ACC GCT GCG ATG GGC TGG TTC CGC CAG GCT CCA GGG AAG GAG CGT GAG TTT GTA GCA GCT ATT AGG TG G AGT GGT GGT AGC GCA TAC TAT GCA GAC TCC GTG AAG GGC CGA TTC AAC ATC TCC AGA GAC AAG GCC AAG AAC ACG GTA TAT CTG CAA ATG AAC AGC CTG AAA T AT GAG GAC ACG GCC GTT TAT TAC TGT GCA CAA ACG CAT TAT GTT TCT TAT CTC TTT AGC GAC TAT GCC ACT TGG CCT TAT GAC TAC TGG GGC CAG GGG ACC CAG GT C ACC GTC TCC TCC TAA

H11-H4 L106W:

CAG GTG CAA CTG GTC GAG TCT GGG GGA GGA TTG ATG CAG GCT GGG GGC TCT C TG AGA CTC TCC TGT GCA GTC TCT GGA CGC ACC TTC AGT ACC GCT GCG ATG GGC TGG TTC CGC CAG GCT CCA GGG AAG GAG CGT GAG TTT GTA GCA GCT ATT AGG TG G AGT GGT GGT AGC GCA TAC TAT GCA GAC TCC GTG AAG GGC CGA TTC AAC ATC TCC AGA GAC AAG GCC AAG AAC ACG GTA TAT CTG CAA ATG AAC AGC CTG AAA T AT GAG GAC ACG GCC GTT TAT TAC TGT GCA CAA ACG CAT TAT GTT TCT TAT CTC

TGG AGC GAC TAT GCC ACT TGG CCT TAT GAC TAC TGG GGC CAG GGG ACC CAG GT
C ACC GTC TCC TCC TAA

SText5 Amino acid sequence and data of the expressed spike protein RBDwild type:

MFVFLVLLPLVSSQRVQPTESIVRFPNITNLCPFGEVFNATRFASVYAWNRKRISNCVADYS
VLYNSASFSTFKCYGVSPTKLNDLCFTNVYADSFVIRGDEVRQIAPGQTGKIADYNYKLPD
DFTGCVIAWNSNNLDSKVGGNYNYLYRLFRKSNLKPFERDISTEIYQAGSTPCNGVEGFNC
YFPLQSYGFQPTNGVGYQPYRVVVLSFELLHAPATVCGPKKSTNLVKNKCVNFHHHHHH

SText6 Amino acid sequence and data of the expressed spike protein RBD delta variant:

MFVFLVLLPLVSSQRVQPTESIVRFPNITNLCPFGEVFNATRFASVYAWNRKRISNCVADYS
VLYNSASFSTFKCYGVSPTKLNDLCFTNVYADSFVIRGDEVRQIAPGQTGKIADYNYKLPD
DFTGCVIAWNSNNLDSKVGGNYNYRYRLFRKSNLKPFERDISTEIYQAGSKPCNGVEGFNC
YFPLQSYGFQPTNGVGYQPYRVVVLSFELLHAPATVCGPKKSTNLVKNKCVNFHHHHHH

SText7 Amino acid sequence of the ecotin fusion protein:

MKTILPAVLFAAFATTSAWAAESVQPLEKIAPYPQAEKGMKRQVIQLTPQEDESTLKVELLI
GQTLEVDCNLHRLGGKLENKTLEGWGYDYYVFDKVSSPVSTMMACPDGKKEKKFVTAYL
GDAGMLRYNSKLPIVVYTPDNVDVKYRVWKAEEKIDNAVVRGSTSGSGHHHHHHSAGLV
PR|GS

SText8 Amino acid sequence of the expressed nanobodies (residual amino acids from the cleaving are underlined):

ab8:

<u>GS</u>QVQLVESGGGLVQPGGSLRLSCAASGFTFDDYAMSWVRQAPGKGLEWIGRMYNNGR
TSYNPSLKSLVTISRDNSKNTLYLQMNSLRAEDTATYYCARDNLGYRPSENLYGMDVWGQ
GTQVTVSS

ab8 E127W:

<u>GS</u>EVQLVESGGGLVQPGGSLRLSCAASGFTFDDYAMSWVRQAPGKGLEWIGRMYNNGR
TSYNPSLKSLVTISRDNSKNTLYLQMNSLRAEDTATYYCARDNLGYRPS**W**NLYGMDVWG
QGTTVTVSS

ab8 S79Y:

<u>GS</u>EVQLVESGGGLVQPGGSLRLSCAASGFTFDDYAMSWVRQAPGKGLEWIGRMYNNGR
T**Y**YNPSLKSLVTISRDNSKNTLYLQMNSLRAEDTATYYCARDNLGYRPSENLYGMDVWG
QGTTVTVSS

ab8 L129F:

<u>GS</u>EVQLVESGGGLVQPGGSLRLSCAASGFTFDDYAMSWVRQAPGKGLEWIGRMYNNGR
TSYNPSLKSLVTISRDNSKNTLYLQMNSLRAEDTATYYCARDNLGYRPSEN**F**YGMDVWG
QGTTVTVSS

H11-H4:

<u>GS</u>QVQLVESGGGLMQAGGSLRLSCAVSGRTFSTAAMGWFRQAPGKEREFVAAIRWSGGS
AYYADSVKGRFTISRDKAKNTVYLQMNSLKYEDTAVYYCAQTHYVSYLLSDYATWPYDY
WGQGTQVTVSS

H11-H4 H100Y:

<u>GS</u>QVQLVESGGGLMQAGGSLRLSCAVSGRTFSTAAMGWFRQAPGKEREFVAAIRWSGGS
AYYADSVKGRFTISRDKAKNTVYLQMNSLKYEDTAVYYCAQT**Y**YVSYLLSDYATWPYDY
WGQGTQVTVSS

H11-H4 V102Y:

<u>GS</u>QVQLVESGGGLMQAGGSLRLSCAVSGRTFSTAAMGWFRQAPGKEREFVAAIRWSGGS
AYYADSVKGRFTISRDKAKNTVYLQMNSLKYEDTAVYYCAQTH**Y**YSYLLSDYATWPYDY
WGQGTQVTVSS

H11-H4 L106F:

<u>GS</u>QVQLVESGGGLMQAGGSLRLSCAVSGRTFSTAAMGWFRQAPGKEREFVAAIRWSGGS
AYYADSVKGRFTISRDKAKNTVYLQMNSLKYEDTAVYYCAQTHYVSYL**F**SDYATWPYDY
WGQGTQVTVSS

H11-H4 L106W:

<u>GS</u>QVQLVESGGGLMQAGGSLRLSCAVSGRTFSTAAMGWFRQAPGKEREFVAAIRWSGGS
AYYADSVKGRFTISRDKAKNTVYLQMNSLKYEDTAVYYCAQTHYVSYL**W**SDYATWPYDY
WGQGTQVTVSS

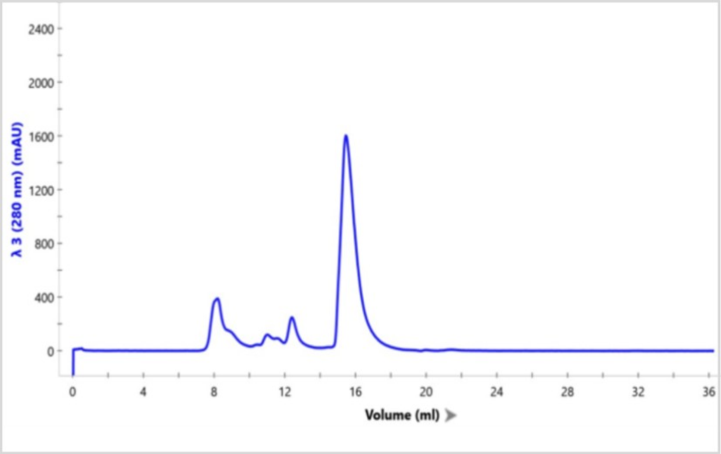

SFig1 Size-exclusion chromatograms as the last purification step of the expression of the ab8 E127W mutant nanobody. The target protein appears at the fraction around 16 ml in accordance to its size.

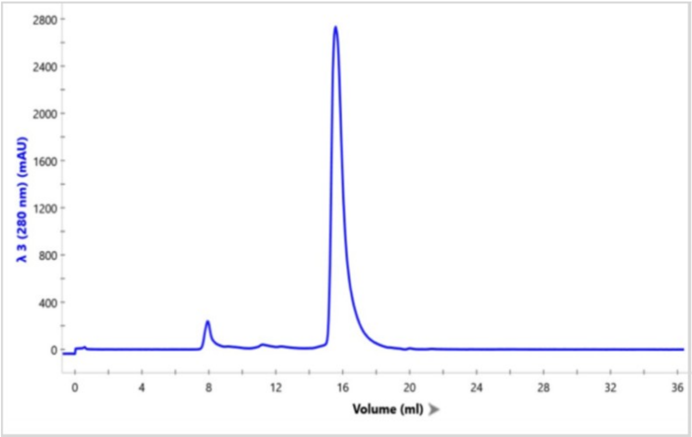

SFig2 Size-exclusion chromatograms as the last purification step of the expression of the H11-H4 H100Y mutant nanobody. The target protein appears at the fraction around 16 ml in accordance to its size.

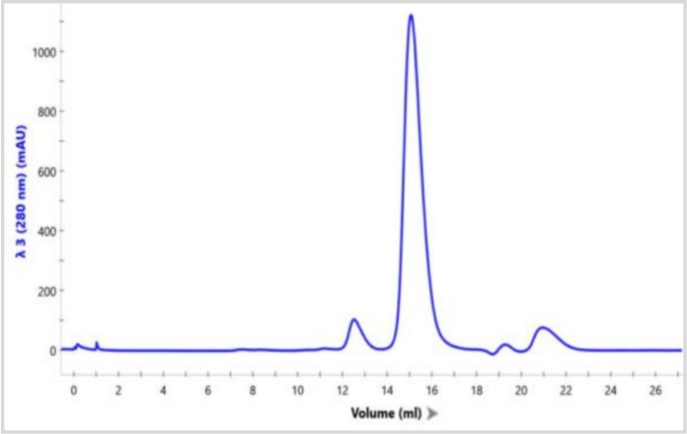

SFig3 Size-exclusion chromatograms as the last purification step of the expression of the H11-H4 V102Y mutant nanobody. The target protein appears at the fraction around 16 ml in accordance to its size.

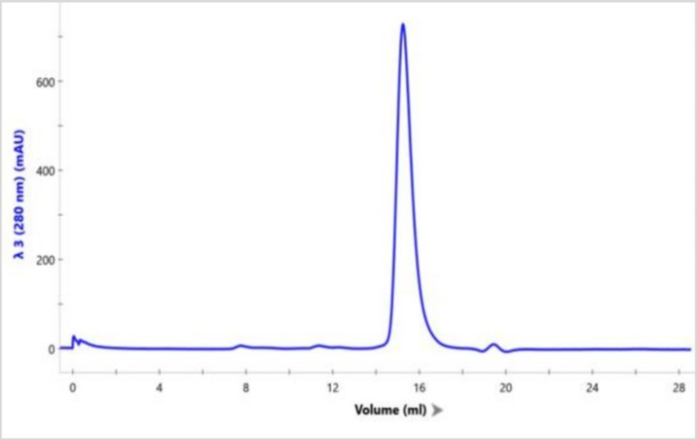

SFig4 Size-exclusion chromatograms as the last purification step of the expression of the H11-H4 L106F mutant nanobody. The target protein appears at the fraction around 16 ml in accordance to its size.

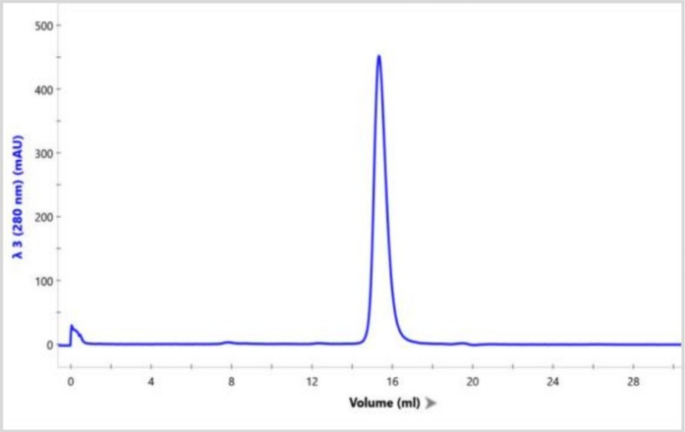

SFig5 Size-exclusion chromatograms as the last purification step of the expression of the H11-H4 L106W mutant nanobody. The target protein appears at the fraction around 16 ml in accordance to its size.

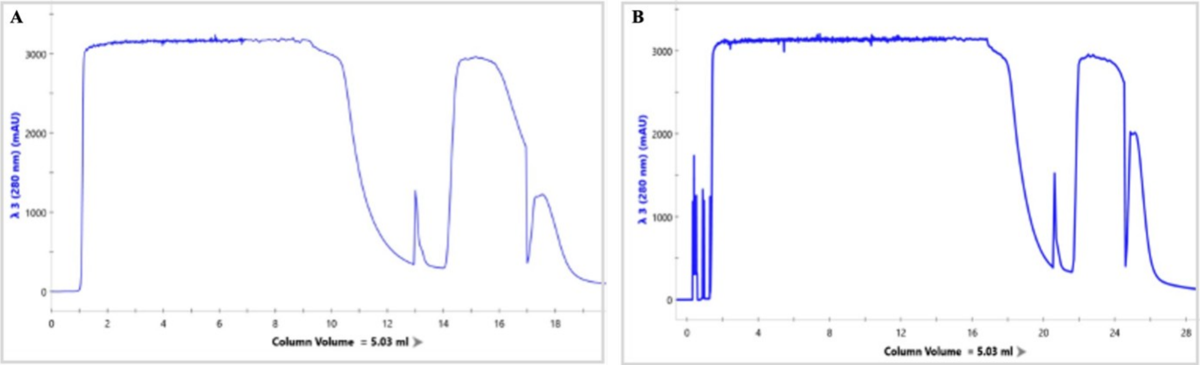

SFig6 The Ni-IMAC chromatography purification step of the (**A**) ab8 S79Y and the (**B**) ab8 L129F mutant nanobodies.

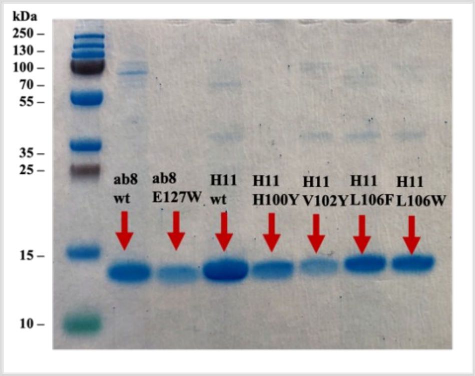

SFig7 Comparison of the purified wild type and mutant nanobodies (ab8 wt, ab8 E127W, H11-H4 wt, H11-H4 H100Y, H11-H4 V102Y, H11-H4 L106F, H11-H4 L106W) by SDS-PAGE. Each sample contains 2.5 µl of sample mixed with 2x SDS loading dye.

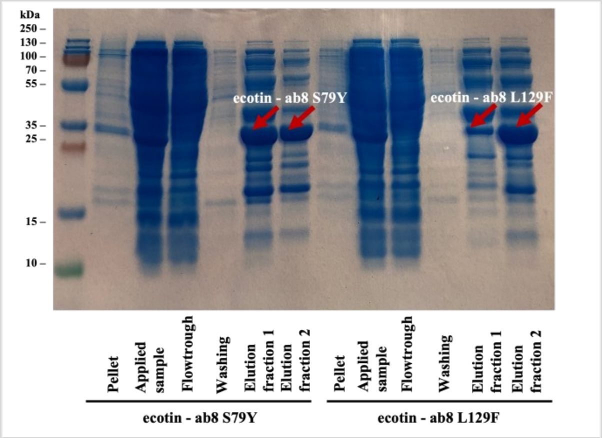

SFig8 Comparison of the different fractions of the mutant nanobodies ab8 S79Y and ab8 L129F from the Ni-IMAC purification step by SDS-PAGE. Each sample contains 2.5 µl of sample mixed with 2x SDS loading dye.

mutant nanobody	yield (mg/L exp.)
ab8 E127W	2.20
ab8 S79Y	-
ab8 L129F	-
H11-H4 L106W	0.60
H11-H4 L106F	0.75
H11-H4 V102Y	0.22
H11-H4 H100Y	1.00

STable1 The list of the expression yields (mg/L of expression medium) for the mutant nanobodies.

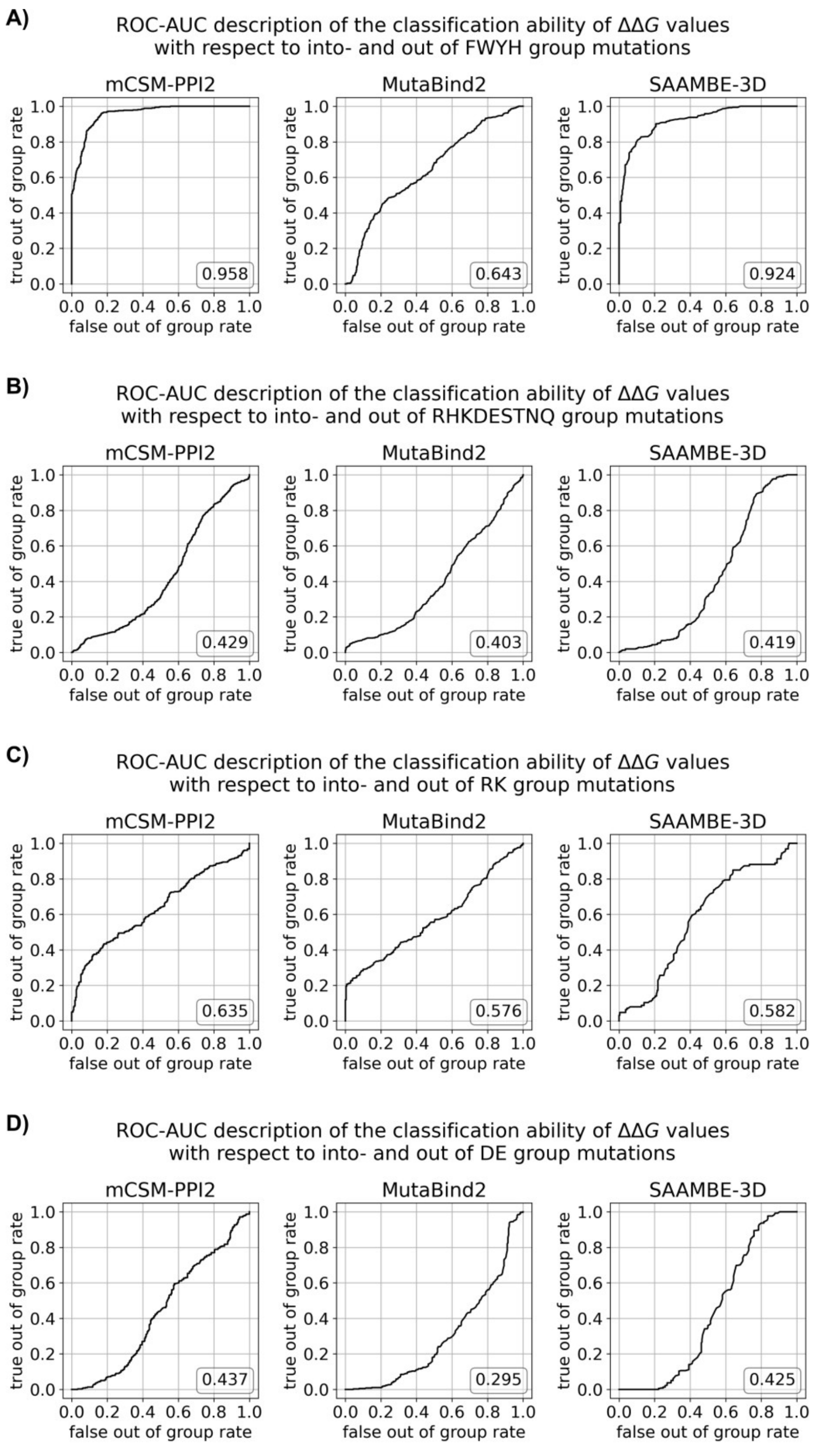

SFig9 Separation ability of ΔΔG regarding into- and out of group mutations for a given residue grouping in the form of receiver operating characteristic (ROC) curves. These curves are depicted separately for each predictor. At the bottom right of each curve the area under the curve (AUC) value is depicted. Greater deviation from the 45° diagonal line indicates better separation. Groupings based on aromaticity (residues FWYH, panel **A**), polarity (residues RHKDESTNQ, panel **B**), positivity (residues RK, panel **C**) and negativity (residues DE, panel **D**) are considered here.

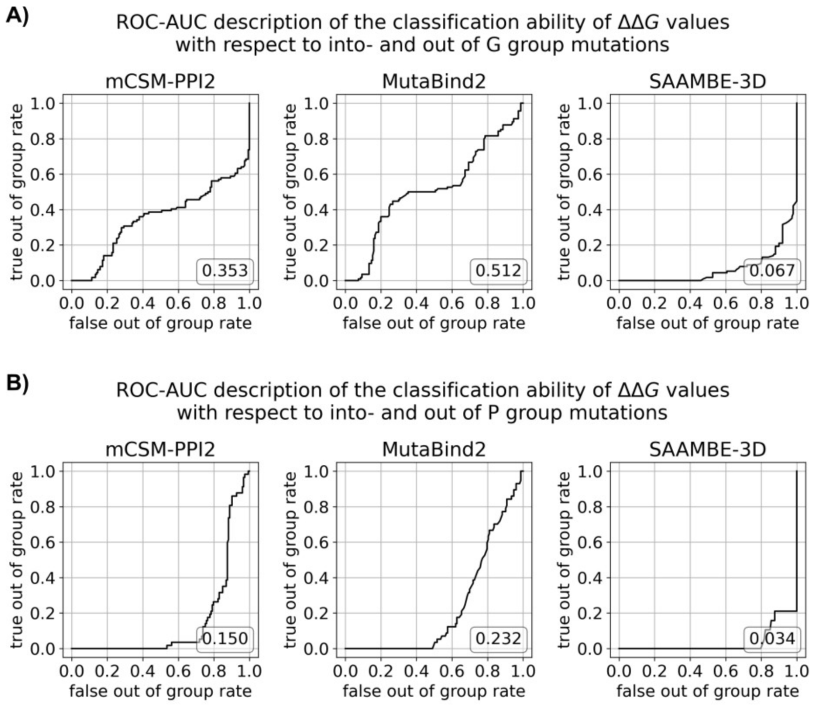

SFig10 Separation ability of ΔΔG regarding into- and out of group mutations for a given residue grouping in the form of receiver operating characteristic (ROC) curves. These curves are depicted separately for each predictor. At the bottom right of each curve the area under the curve (AUC) value is depicted. Greater deviation from the 45° diagonal line indicates better separation. Single residue groups of glycine (panel **A**) and proline (panel **B**) are considered here.

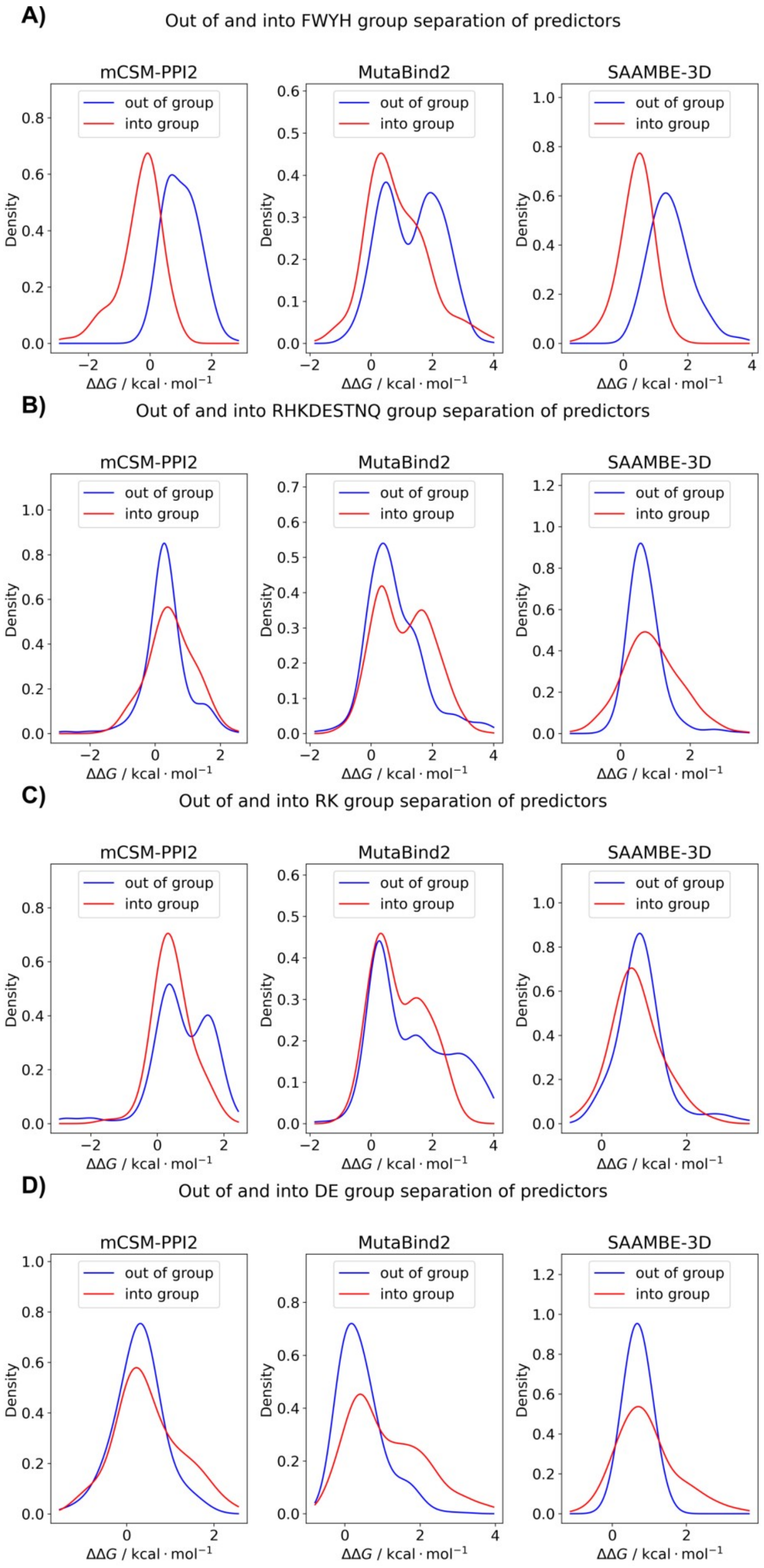

SFig11 ΔΔG densities corresponding to different residue groupings and different predictors. Blue curves show the ΔΔG densities when a residue that is part of the group is mutated to a residue that is not part of the group ("out of group" mutations). Red curves show the ΔΔG densities belonging to mutations that change a non-group residue to an in-group residue ("into group" mutations).

Groupings based on aromaticity (residues FWYH, panel **A**), polarity (residues RHKDESTNQ, panel **B**), positivity (residues RK, panel **C**) and negativity (residues DE, panel **D**) are considered here.

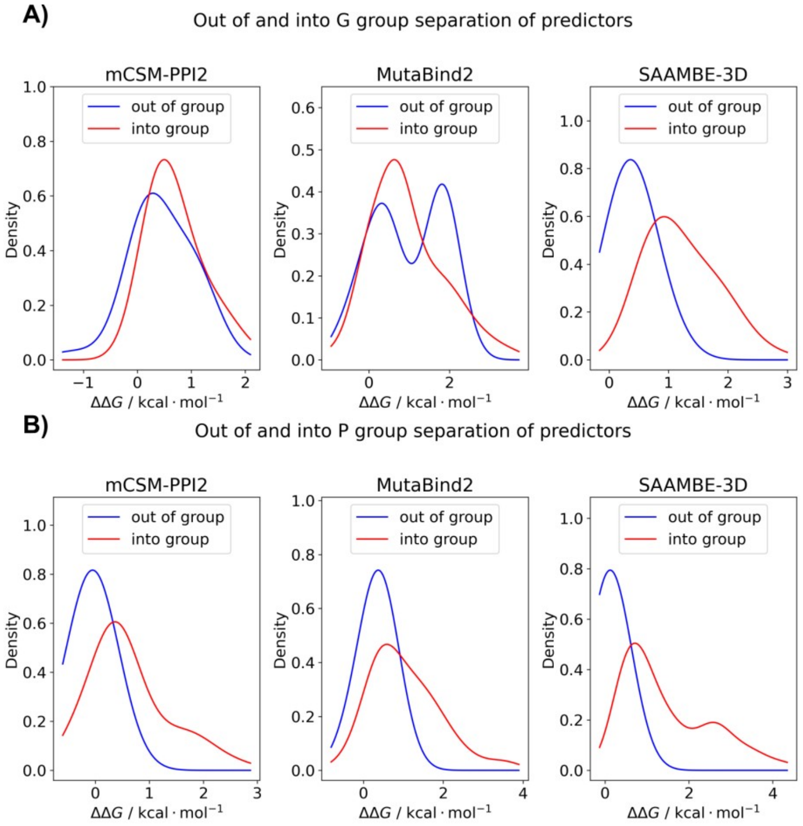

SFig12 ΔΔG densities corresponding to different residue groupings and different predictors. Blue curves show the ΔΔG densities when a residue that is part of the group is mutated to a residue that is not part of the group ("out of group" mutations). Red curves show the ΔΔG densities belonging to mutations that change a non-group residue to an in-group residue ("into group" mutations). Single residue groups of glycine (panel **A**) and proline (panel **B**) are considered here.

SText9 Shifting the focus to individual mutations, the mutation sets for a given nanobody and S-RBD variant pair were ordered according to the $\Delta\Delta G$ values of the mutations within the set. Then, we looked at the best three and worst three mutations in this ordering (**STable2**). It can be seen that in the case of mCSM-PPI2, all the best 3-3 mutations are into aromatic mutations, which is a behavior not observed for MutaBind2 and SAAMBE-3D, favoring into aromatic mutations in 13/27 and 20/27 ratios, respectively. Best mutation site agreement is only partially present among the predictors: in the case of H11-H4, the position L106 is the only mutation position present for all predictors. However, all other suggested mutation positions are sequentially close positions to L106, namely H100, V102 and L105. In Nb20 site A51 is a preferred site for all predictors. Site R31 appears in suggested mutations by mCSM-PPI2 and MutaBind2, but not by SAAMBE-3D, and site A29 is enumerated by MutaBind2 and SAAMBE-3D, but not by mCSM-PPI2. Finally, in the case of ab8, site S79 is strongly suggested for mutation by all predictors, even agreeing that the optimal target residue is Tyr.

	Nanobody	WT S-RBD	S-RBD variant Δ_1	S-RBD variant Δ_2
mCSM-PPI2	H11-H4	V102Y, L106F, L106W, R52Q, Y101D, R52P	V102Y, L106F, L106W, Y101E, R52Q, R52P	V102Y, L106W, L106F, Y101D, Y101E, R52P
	Nb20	R97W, R31W, A51Y, Y104E, R31D, R31P	R31W, R97W, R31Y, R31V, R31Q, R31P	R31W, R97W, R31Y, R31Q, R31P, Y104E
	ab8	R71W, E127W, S79Y, R71P, Y73D, Y73P	R71W, G131Y, S79Y, R71P, Y73D, Y73P	R71W, E127W, G131Y, R71P, Y73D, Y73P
MutaBind2	H11-H4	H100F, L106P, H100Y, R52Y, R52I, R52W	H100K, H100R, H100Q, R52V, R52P, R52W	H100R, H100Q, H100S, R52E, R52D, R52W
	Nb20	R31Y, A51W, H30Y, R97H, R97F, R97Y	H30Y, A51W, A29N, R97H, R97P, R97F	R31Y, H30Y, A29F, Y104T, R97F, Y104P
	ab8	S79Y, S79F, D51S, R71W, Y73D, R71G	S79Y, S79H, S79F, R71C, R71P, R71E	S79Y, N128Y, S79H, R71Q, R71D, R71C
SAAMBE-3D	H11-H4	L106Y, L106F, L106T, Y104D, Y104P, L105P	L106Y, L106T, L105Y, R52P, Y104P, L105P	L106Y, L105Y, L106T, Y104D, Y104P, L105P
	Nb20	A51F, A51N, A51H, Y104A, R31P, Y104P	A102F, A29F, A51F, R97P, R31P, Y104P	A29F, A29Y, A29H, Y104A, R31P, Y104P
	ab8	S79Y, R124K, S79F, Y73P, R77P, R71P	S79Y, R124K, S79F, L121P, R77P, R71P	S79Y, R124K, S79F, L121P, R77P, R71P

STable2 Nanobody mutations with the three smallest (green) and three largest (red) predicted $\Delta\Delta G$ values for all S-RBD subvariants.

SText10 The effect of mutations on the S-RBD, i.e. moving from the WT to either the Δ_1 or Δ_2 subvariants, was investigated by calculating the corresponding $\Delta\Delta\Delta G$ values. For a given predictor, nanobody, and nanobody mutation the $\Delta\Delta\Delta G$ value of moving from the WT S-RBD to the subvariant (SV) is:

$$\Delta\Delta\Delta G(SV) = \Delta\Delta G(SV) - \Delta\Delta G(WT)$$

The most affected individual nanobody mutants by moving from the WT to the delta variants were also collected (**STable3**). Rationally, mutants with really large or small $\Delta\Delta\Delta G$ values are expected to be close to the Δ_1 or Δ_2 mutation sites, namely close to L452R and T478K in the Δ_1 variant, while close to K417N, L452R and T478K in the Δ_2 variant (which differentiate them from the WT).

Negative $\Delta\Delta\Delta G$-valued mutations at positions R52, S57, Y101, V102, W112 in H11-H4 are the ones most affected by the introduction of the delta mutations into the S-RBD (see **SFig13/A**). These sites surround position L452 of the S-RBD, which explains the changes in the predicted $\Delta\Delta G$ values of their corresponding mutations. Position S57 is the furthest away from L452 with a distance of 13.7 Å, almost as far as it is from T478 (14.6 Å). The S57Q mutation is predicted to be largely affected only by MutaBind2, which is the only ML model that corrects the input and mutated structure with a short energy minimization step. It can be safely assumed that the effects of the delta mutations on S57Q can only be seen correctly through a modified complex structure. In the case of Nb20, sites G28, A29, R31, F35, A102 are the ones that are the most affected by WT-Δ_1 or WT-Δ_2 changes by the three predictors (see **SFig13/B**). These sites are further away from L452 than the highlighted sites in H11-H4: R31 is 7.9 Å away from L452, which is the closest distance to this set of sites, in contrast to the H11-H4 case, where V102 is the closest to L452 with a distance of 3.8 Å. The site interval A51-M55 in Nb20 is closer to L452 than the residues mentioned above, and mutations at A51 and M55 are indeed present in the table, albeit as mutations with the most positive $\Delta\Delta\Delta G$ values. Finally, in ab8 mutations at sites N74, S79, L121, E127 and G131 are predicted to have the most negative $\Delta\Delta\Delta G$ values (**SFig13/C**). N74 is closest to the S-RBD delta mutation site L452, L121 is closest to K417, E127 and G131 are closest to T478, and S79 is far away from all three delta mutation sites.

	Nanobody	S-RBD variant Δ_1	S-RBD variant Δ_2
mCSM-PPI2	H11-H4	V102R, V102K, V102N, L106D, L106Q, R52N	W112R, W112E, Y101G, L106R, L106Q, L106N
	Nb20	F35W, R31W, F35I, D98R, D98K, N72Y	F35V, F35I, R31Y, D98R, N72Y, D98T
	ab8	N74D, E127R, E127M, W68K, Y130E, W68R	N74D, E127M, G131H, W68R, W68K, Y130E
MutaBind2	H11-H4	V102A, S57Q, V102P, S103N, S103T, L106C	R52Y, R52I, V102D, L106T, L106C, L106A
	Nb20	A29Q, A29T, A29R, A51E, M55W, Y104V	A29Q, A29T, A29I, R31V, A51M, A51E
	ab8	E127R, S79E, L121F, L121R, D119H, R71M	L121E, S79E, L121F, N75K, N75R, R71M
SAAMBE-3D	H11-H4	V102D, V102G, V102S, L105V, L105I, L105A	V102D, V102G, V102S, L105V, L105I, L105A
	Nb20	A102I, A102L, G28L, A51Y, A51H, A51W	A102I, A102L, A102G, A51Y, A51W, A51F
	ab8	G131K, G131R, G131H, N75E, N75K, N75M	G131K, G131R, G131Y, N75E, N75K, N75M

STable3 Nanobody mutations with the three smallest (green) and three largest (red) predicted $\Delta\Delta\Delta G$ values for both delta S-RBD subvariants. These nanobody mutations are the ones that are the most affected by the introduction of the delta subvariant mutations into the S-RBD.

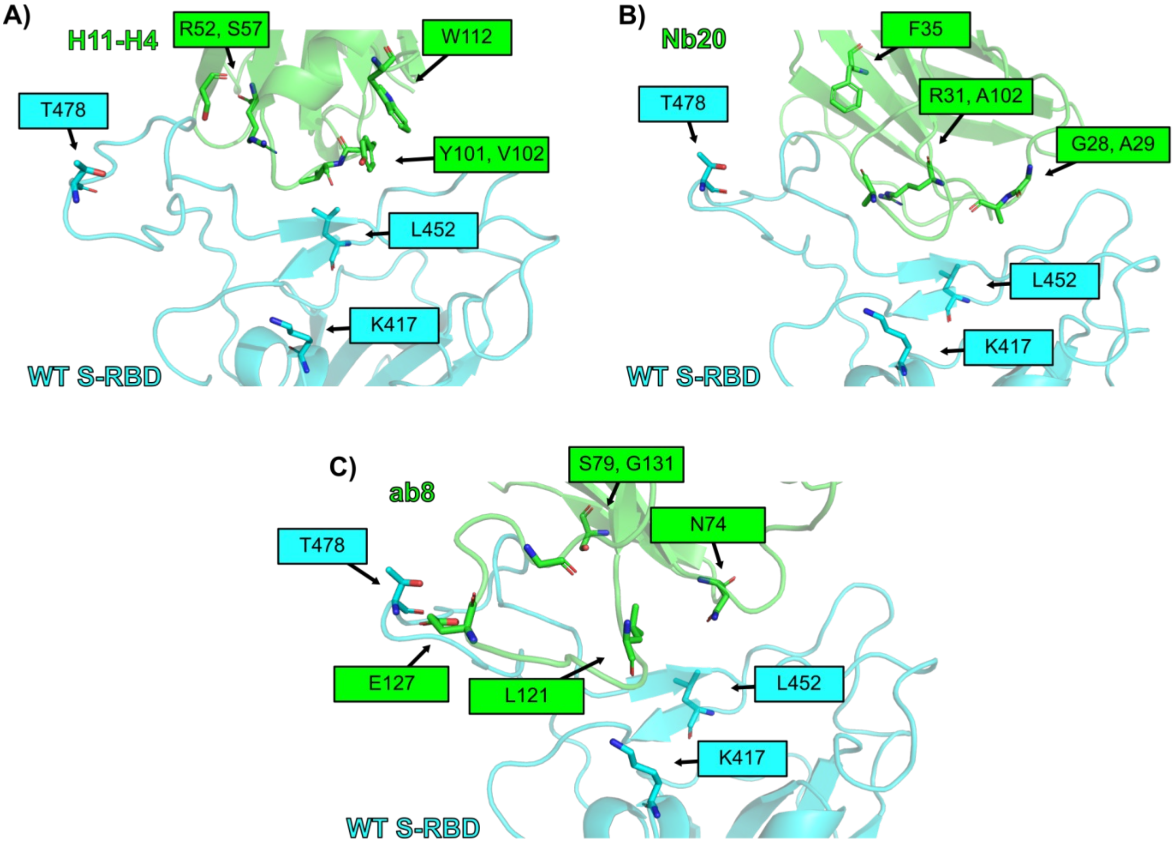

SFig13 The display of the nanobody mutation sites with the most negative ΔΔΔ*G* values according to the three predictors (green residues and boxes). The Δ_1 and Δ_2 mutation sites on the S-RBD are also indicated (cyan residues and boxes). Panels **A**, **B**, and **C** show the residues for nanobodies H11-H4, Nb20 and ab8, respectively. While in H11-H4 almost all sites are close to the mutation site L452, which is present in both Δ_1 and Δ_2, this effect is less pronounced in the case of Nb20, and in the case of ab8 the nanobody mutation sites belong to all three different S-RBD delta mutation sites.

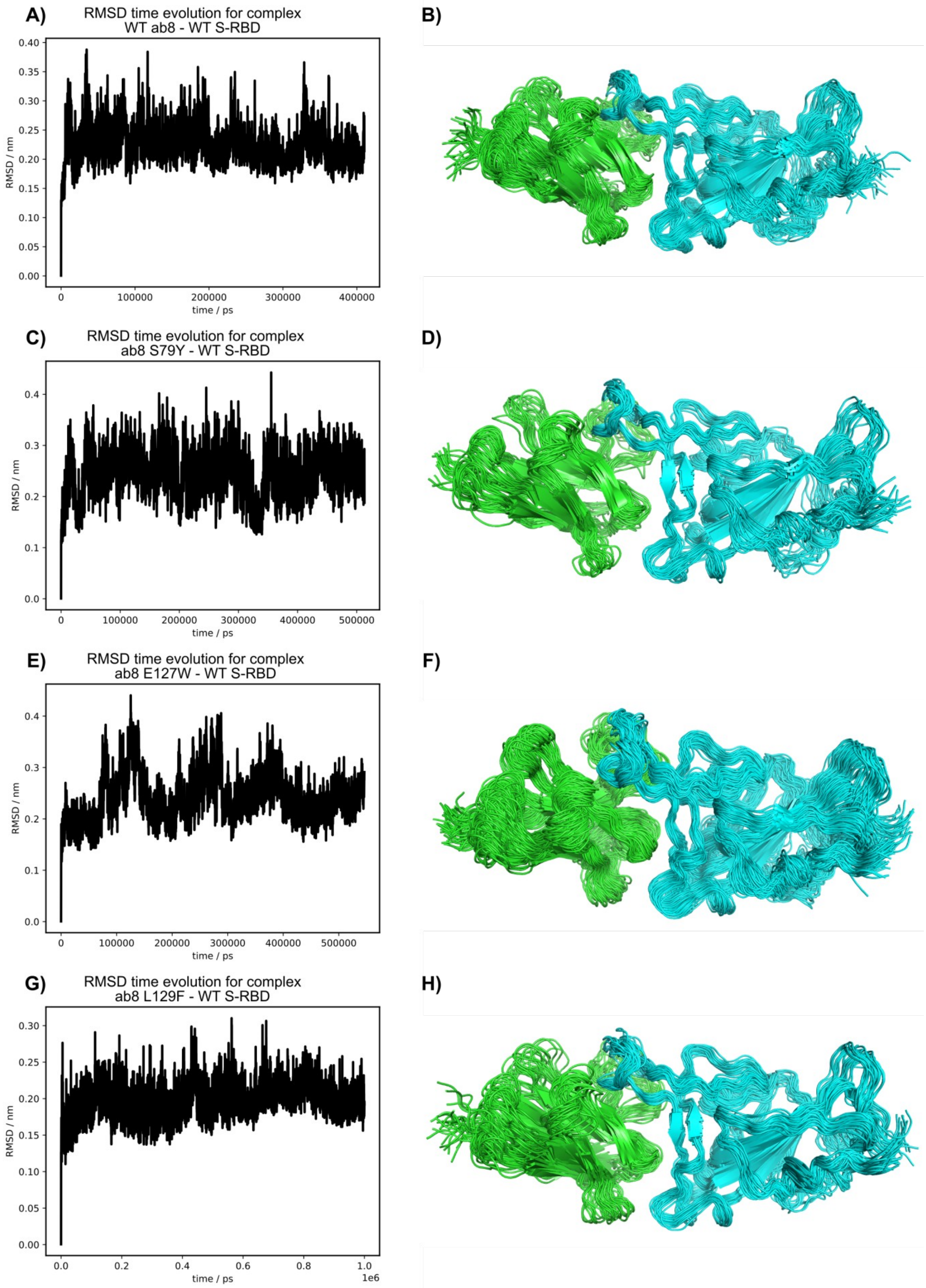

SFig14 RMSD time evolution (panels **A**, **C**, **E** and **G**) and corresponding cluster centers (panels **B**, **D**, **F** and **H**) for different nanobody/S-RBD complexes (WT ab8/WT S-RBD, S79Y ab8/WT S-RBD, E127W ab8/WT S-RBD, L129F ab8/WT S-RBD, respectively).

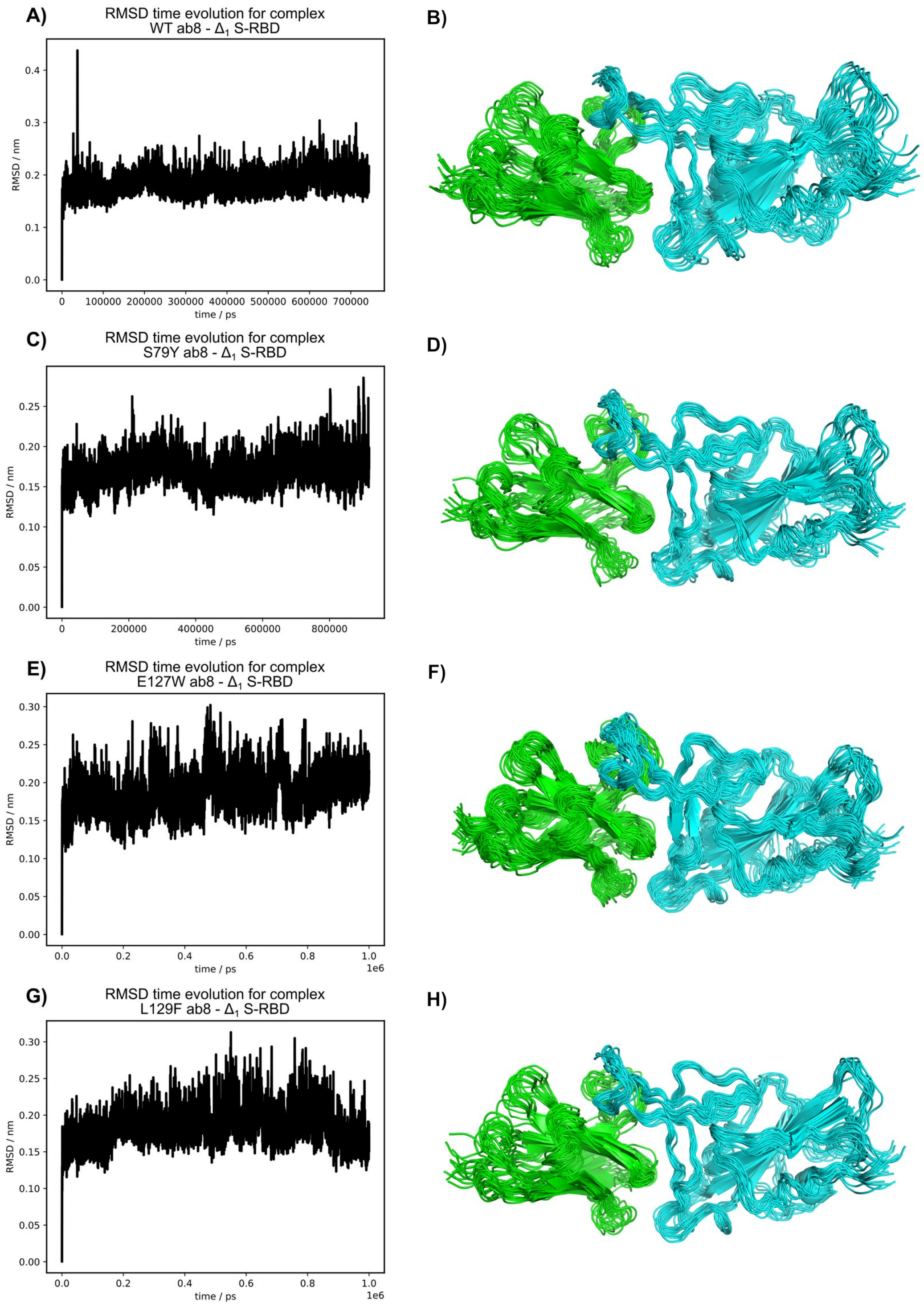

SFig15 RMSD time evolution (panels **A**, **C**, **E** and **G**) and corresponding cluster centers (panels **B**, **D**, **F** and **H**) for different nanobody/S-RBD complexes (WT ab8/Δ_1 S-RBD, S79Y ab8/Δ_1 S-RBD, E127W ab8/Δ_1 S-RBD, L129F ab8/Δ_1 S-RBD, respectively).

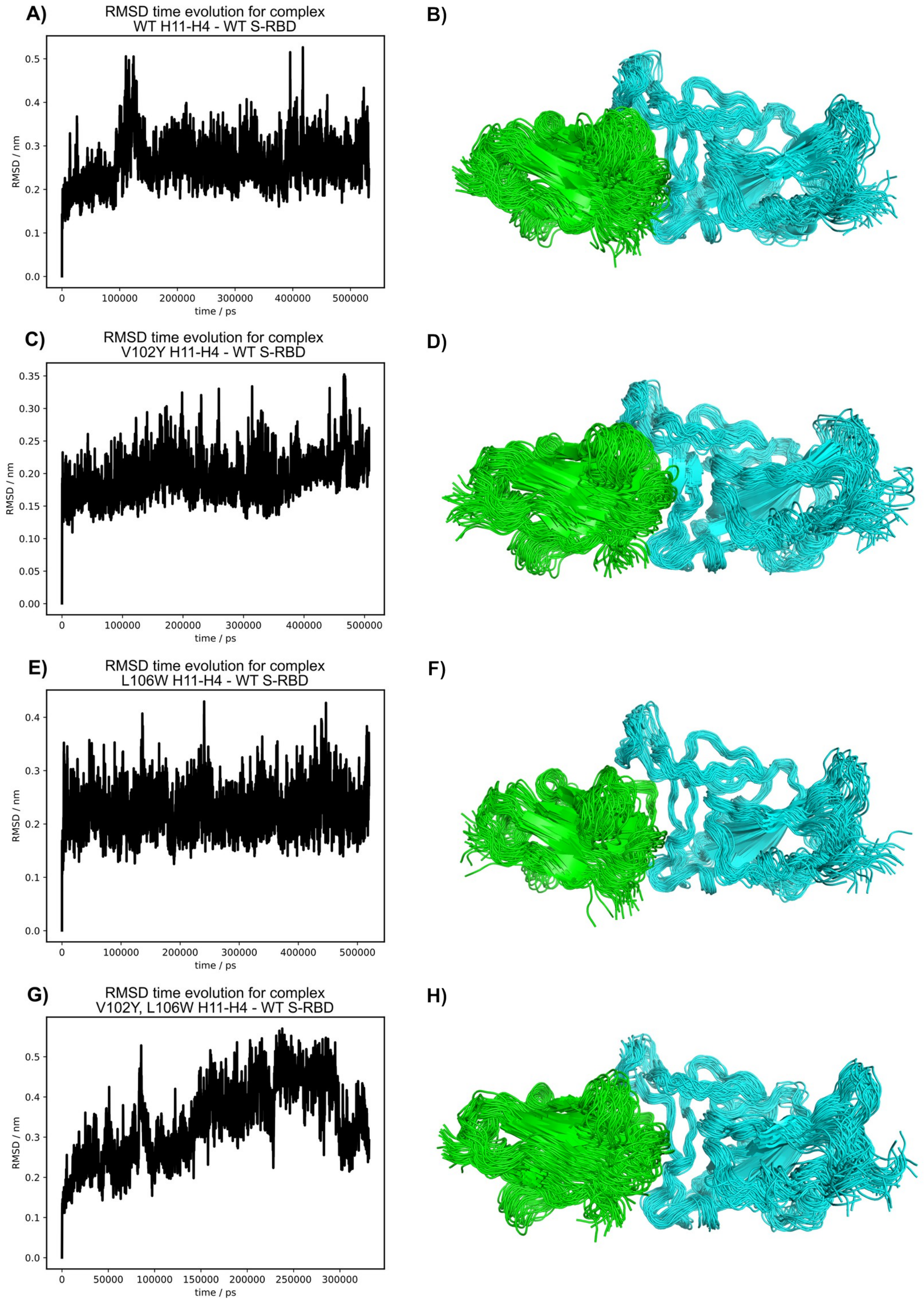

SFig16 RMSD time evolution (panels **A**, **C**, **E** and **G**) and corresponding cluster centers (panels **B**, **D**, **F** and **H**) for different nanobody/S-RBD complexes (WT H11-H4/WT S-RBD, V102Y H11-H4/WT S-RBD, L106W H11-H4/WT S-RBD, V102Y+L106W H11-H4/WT S-RBD, respectively).

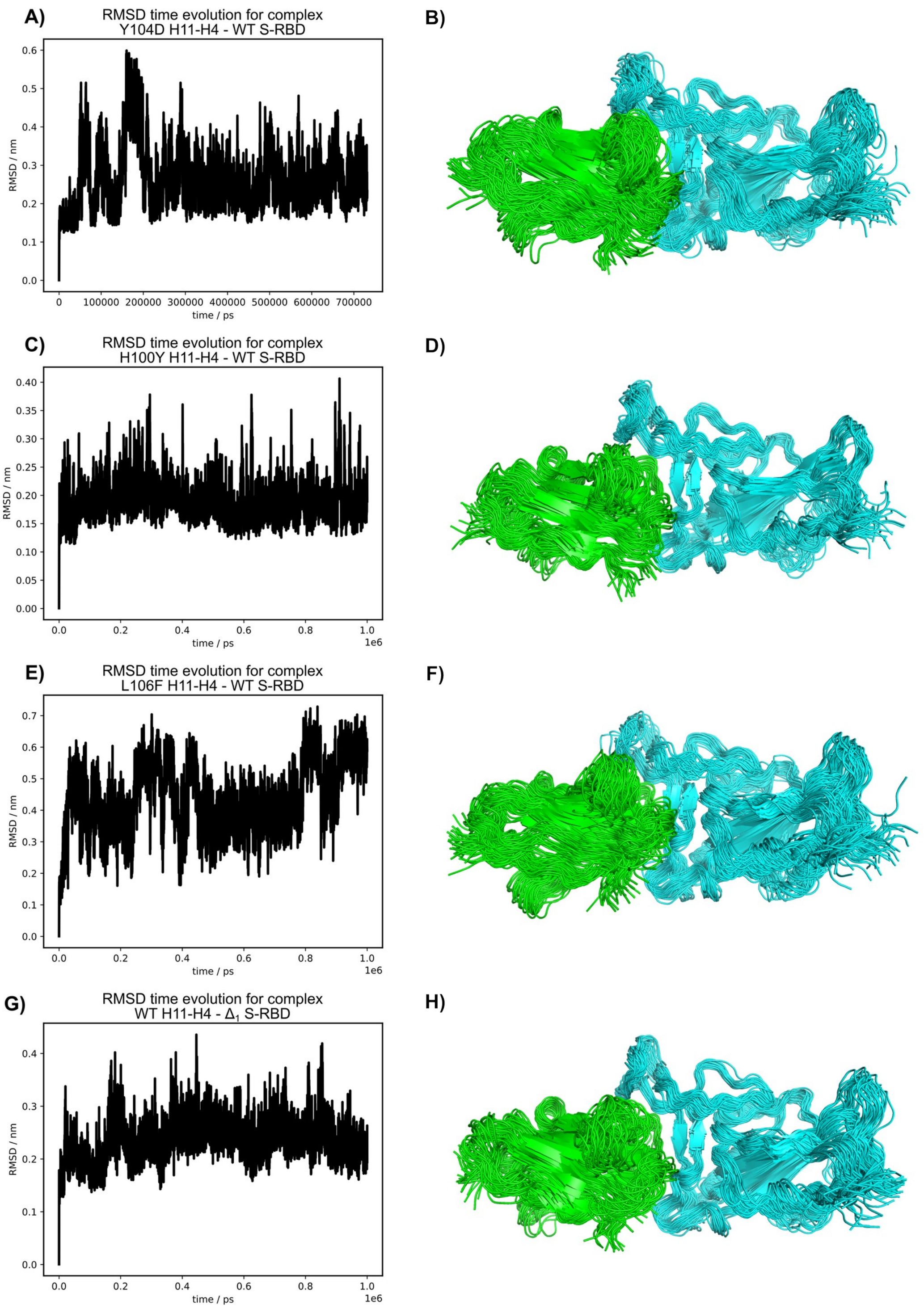

SFig17 RMSD time evolution (panels **A**, **C**, **E** and **G**) and corresponding cluster centers (panels **B**, **D**, **F** and **H**) for different nanobody/S-RBD complexes (Y104D H11-H4/WT S-RBD, H100Y H11-H4/WT S-RBD, L106F H11-H4/WT S-RBD, WT H11-H4/Δ_1 S-RBD, respectively).

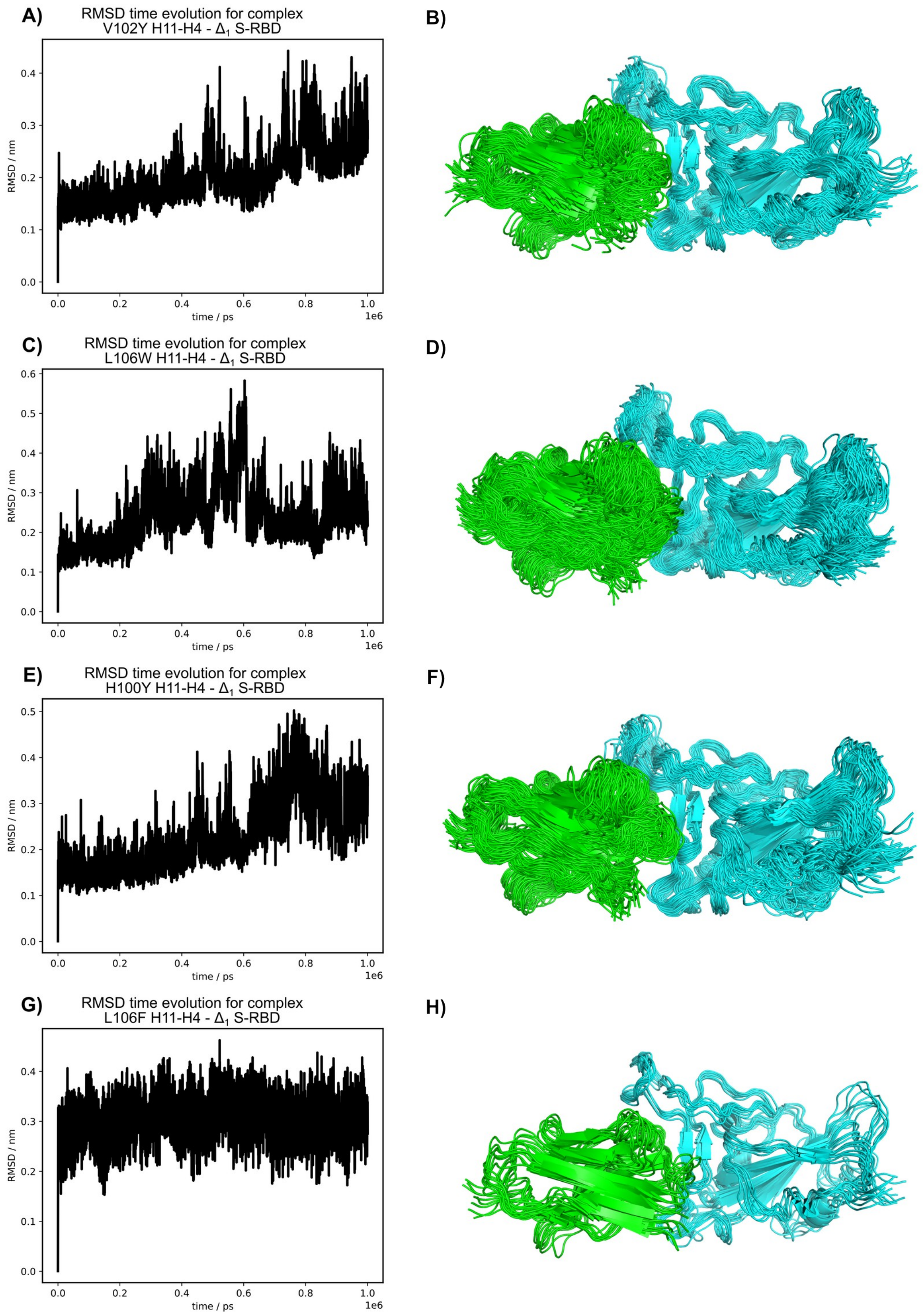

SFig18 RMSD time evolution (panels **A**, **C**, **E** and **G**) and corresponding cluster centers (panels **B**, **D**, **F** and **H**) for different nanobody/S-RBD complexes (V102Y H11-H4/Δ_1 S-RBD, L106W H11-H4/Δ_1 S-RBD, H100Y H11-H4/Δ_1 S-RBD, L106F H11-H4/Δ_1 S-RBD, respectively).

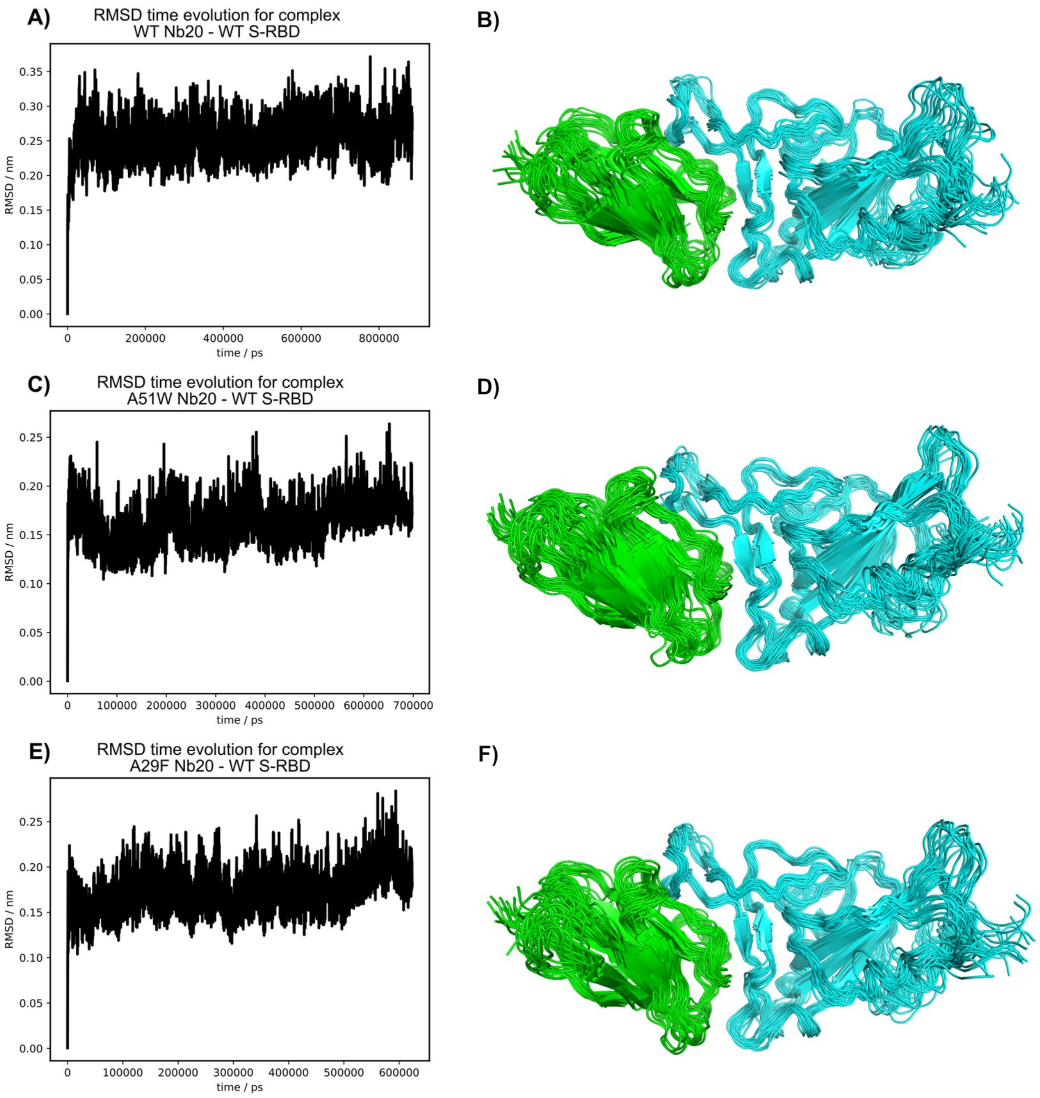

SFig19 RMSD time evolution (panels **A**, **C**, and **E**) and corresponding cluster centers (panels **B**, **D**, and **F**) for different nanobody/S-RBD complexes (WT Nb20/WT S-RBD, A51W Nb20/WT S-RBD, A29F H11-H4/WT S-RBD, respectively).

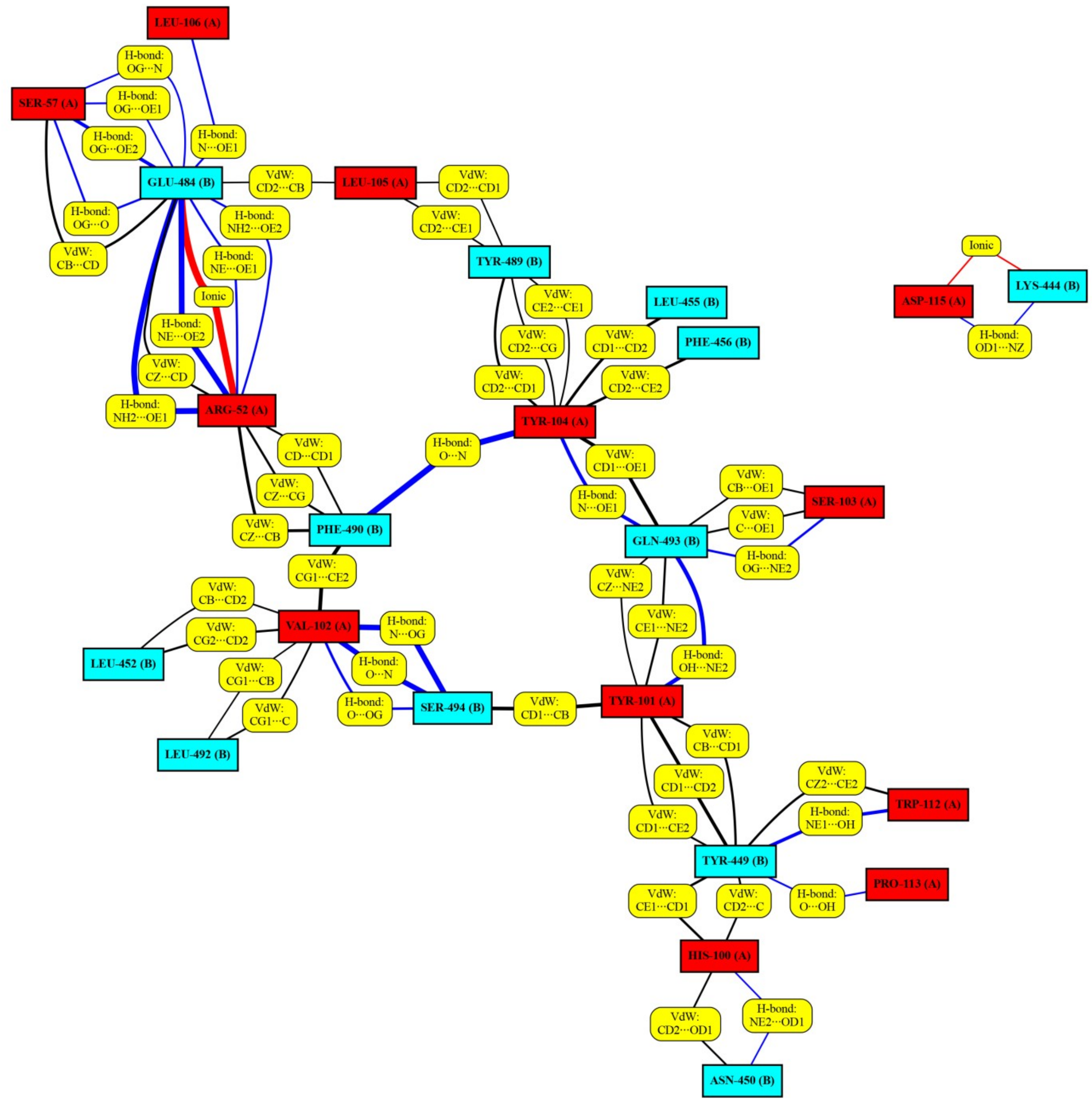

SFig20 Hetero-chain residue-residue interaction network of the WT H11-H4/WT S-RBD complex calculated from its molecular dynamics simulation. Red boxes belong to chain A (H11-H4 nanobody), cyan boxes belong to chain B (WT S-RBD protein), and yellow boxes depict the interaction type and interacting atoms (in the order of chain A side atom, then chain B side atom). Redundantly, edges on this graph are colored blue for H-bonds, black for Van der Waals interactions, red for ionic interactions and green for pi-pi stacking or pi-cation interactions. Interactions that are present more abundantly along the trajectory are depicted with thicker lines.

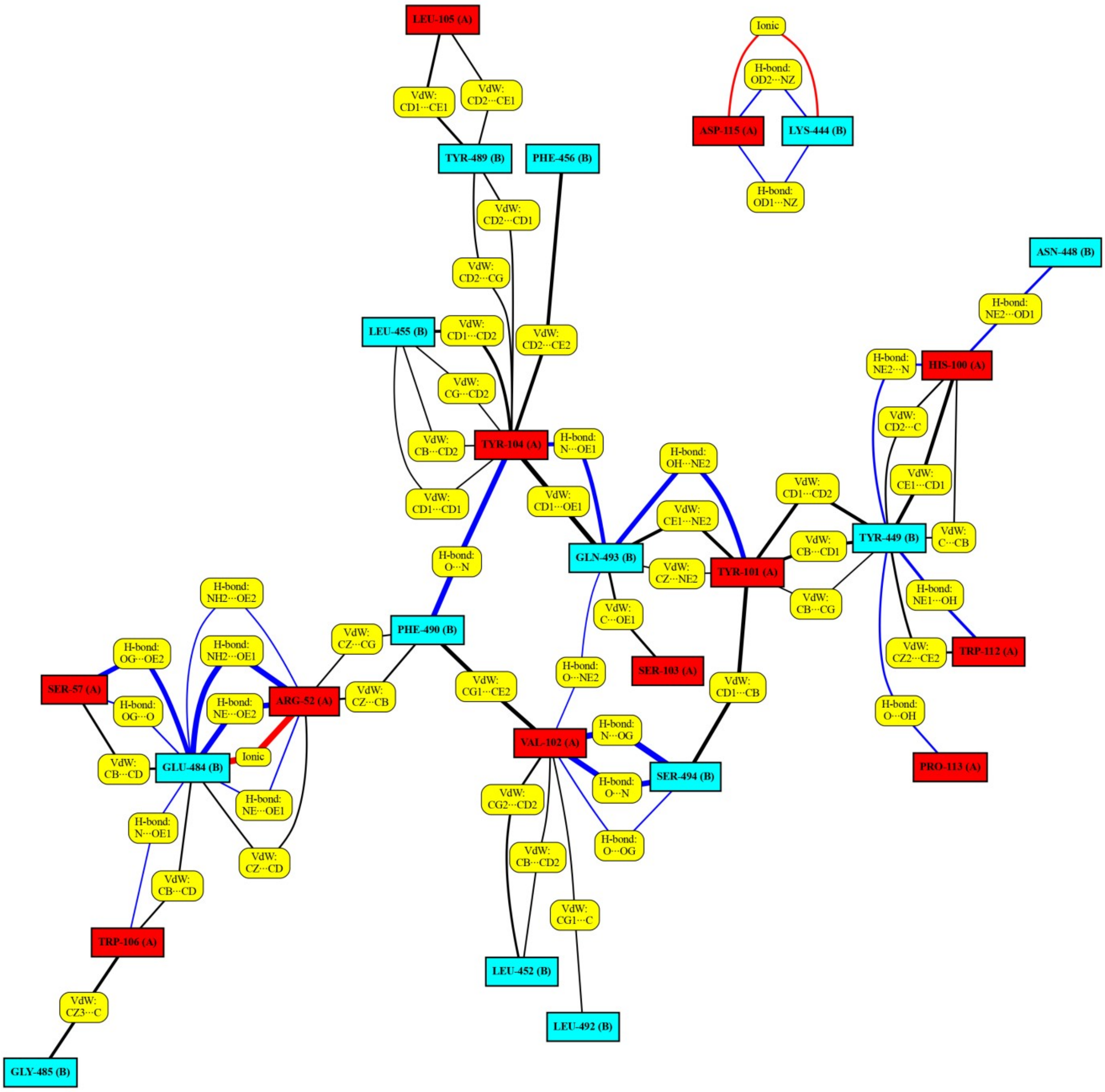

SFig21 Hetero-chain residue-residue interaction network of the L106W mutant H11-H4/WT S-RBD complex calculated from its molecular dynamics simulation. Red boxes belong to chain A (L106W H11-H4 nanobody), cyan boxes belong to chain B (WT S-RBD protein), and yellow boxes depict the interaction type and interacting atoms (in the order of chain A side atom, then chain B side atom). Redundantly, edges on this graph are colored blue for H-bonds, black for Van der Waals interactions, red for ionic interactions and green for pi-pi stacking or pi-cation interactions. Interactions that are present more abundantly along the trajectory are depicted with thicker lines.

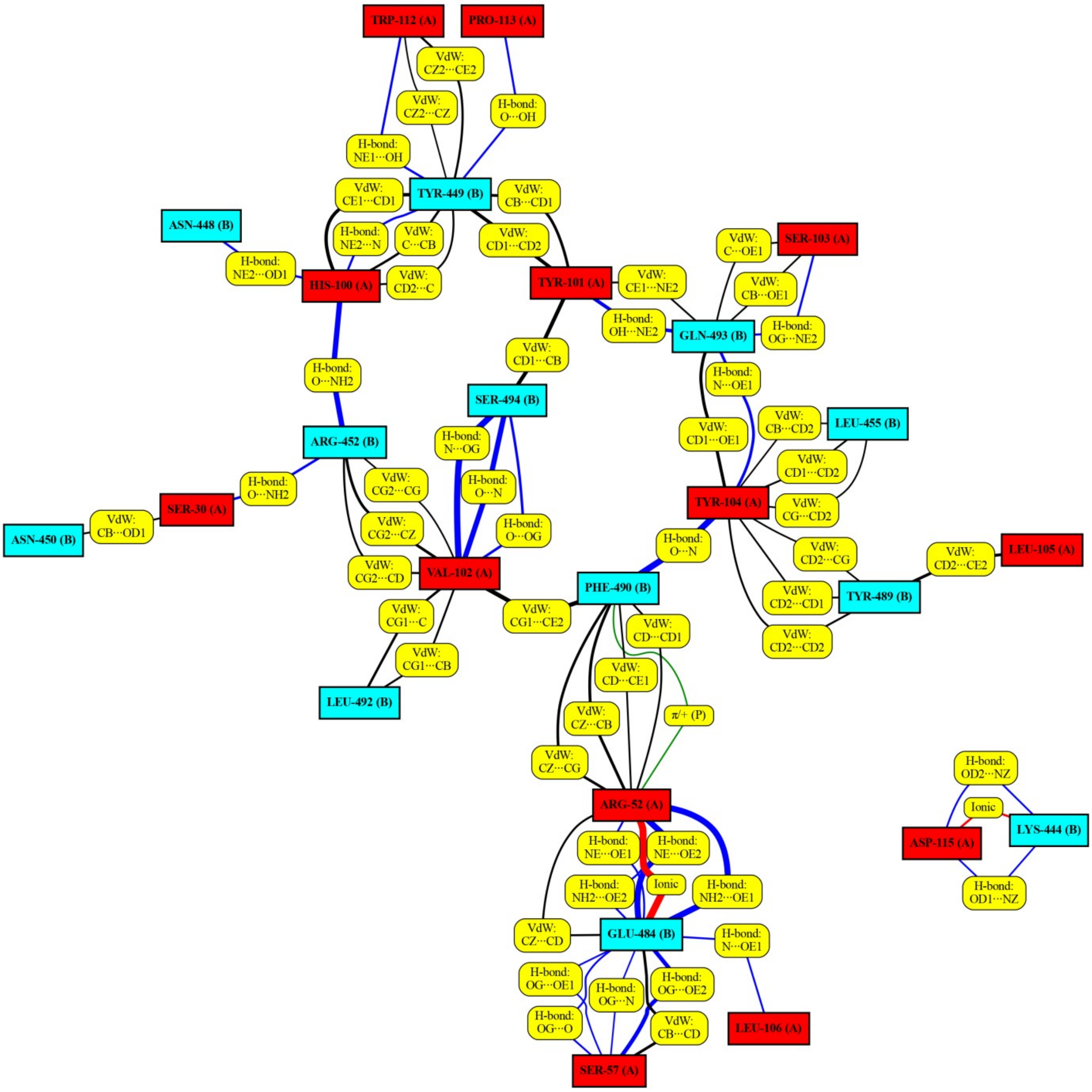

SFig22 Hetero-chain residue-residue interaction network of the WT H11-H4/Δ_1 S-RBD complex calculated from its molecular dynamics simulation. Red boxes belong to chain A (H11-H4 nanobody), cyan boxes belong to chain B (Δ_1 S-RBD protein), and yellow boxes depict the interaction type and interacting atoms (in the order of chain A side atom, then chain B side atom). Redundantly, edges on this graph are colored blue for H-bonds, black for Van der Waals interactions, red for ionic interactions and green for pi-pi stacking or pi-cation interactions. Interactions that are present more abundantly along the trajectory are depicted with thicker lines.

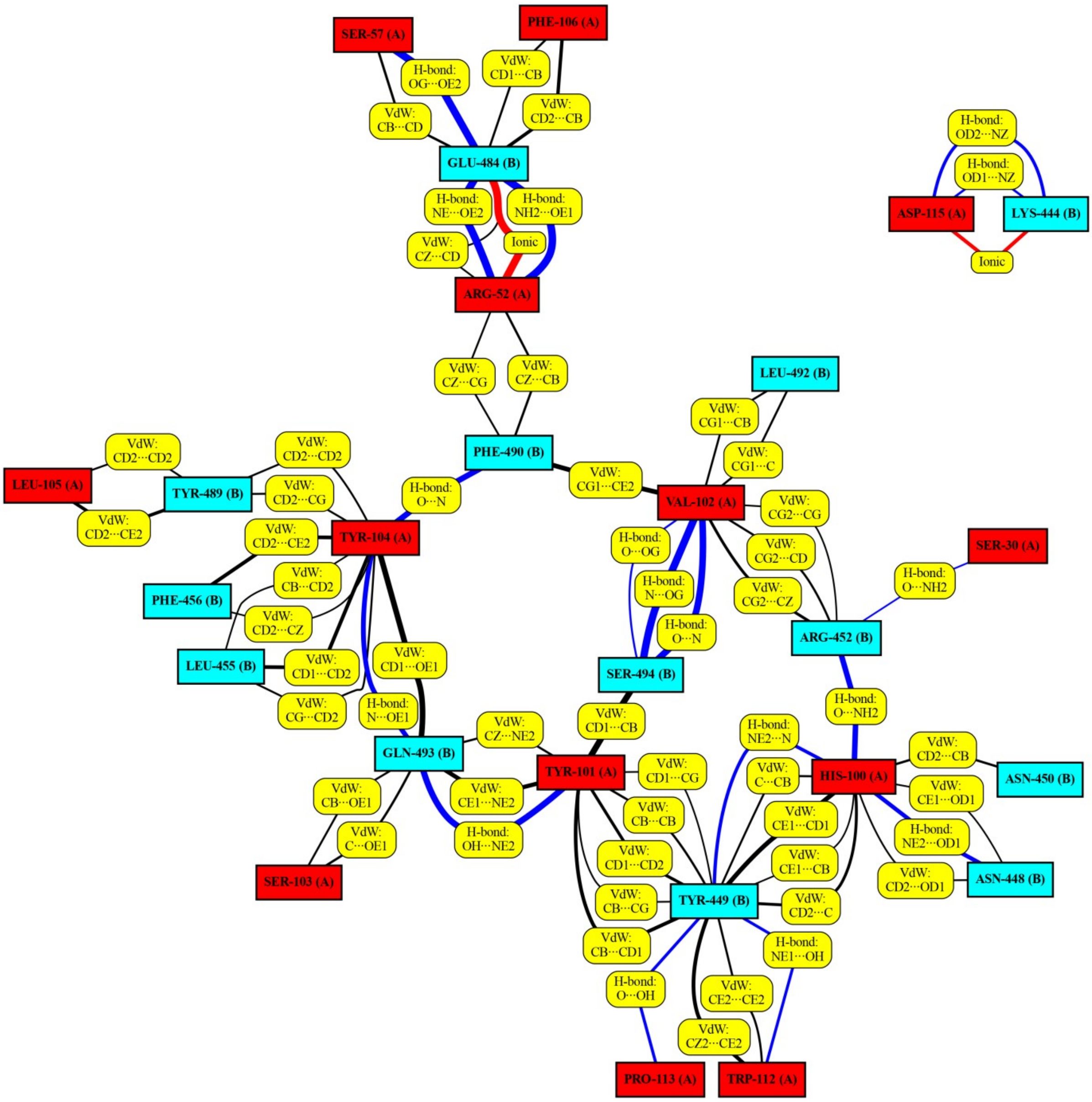

SFig23 Hetero-chain residue-residue interaction network of the L106F mutant H11-H4/Δ_1 S-RBD complex calculated from its molecular dynamics simulation. Red boxes belong to chain A (L106F H11-H4 nanobody), cyan boxes belong to chain B (Δ_1 S-RBD protein), and yellow boxes depict the interaction type and interacting atoms (in the order of chain A side atom, then chain B side atom). Redundantly, edges on this graph are colored blue for H-bonds, black for Van der Waals interactions, red for ionic interactions and green for pi-pi stacking or pi-cation interactions. Interactions that are present more abundantly along the trajectory are depicted with thicker lines.

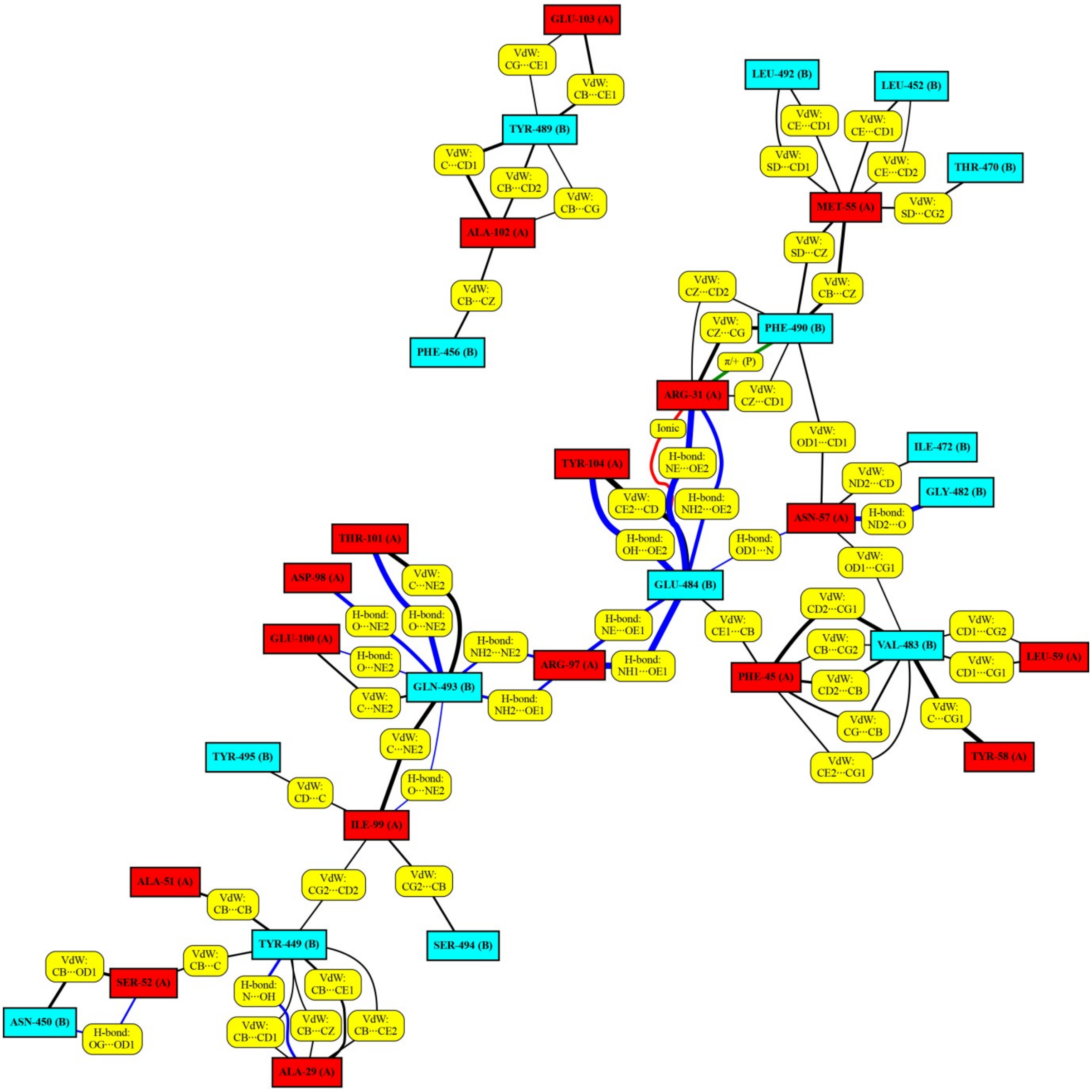

SFig24 Hetero-chain residue-residue interaction network of the WT Nb20/WT S-RBD complex calculated from its molecular dynamics simulation. Red boxes belong to chain A (Nb20 nanobody), cyan boxes belong to chain B (WT S-RBD protein), and yellow boxes depict the interaction type and interacting atoms (in the order of chain A side atom, then chain B side atom). Redundantly, edges on this graph are colored blue for H-bonds, black for Van der Waals interactions, red for ionic interactions and green for pi-pi stacking or pi-cation interactions. Interactions that are present more abundantly along the trajectory are depicted with thicker lines.

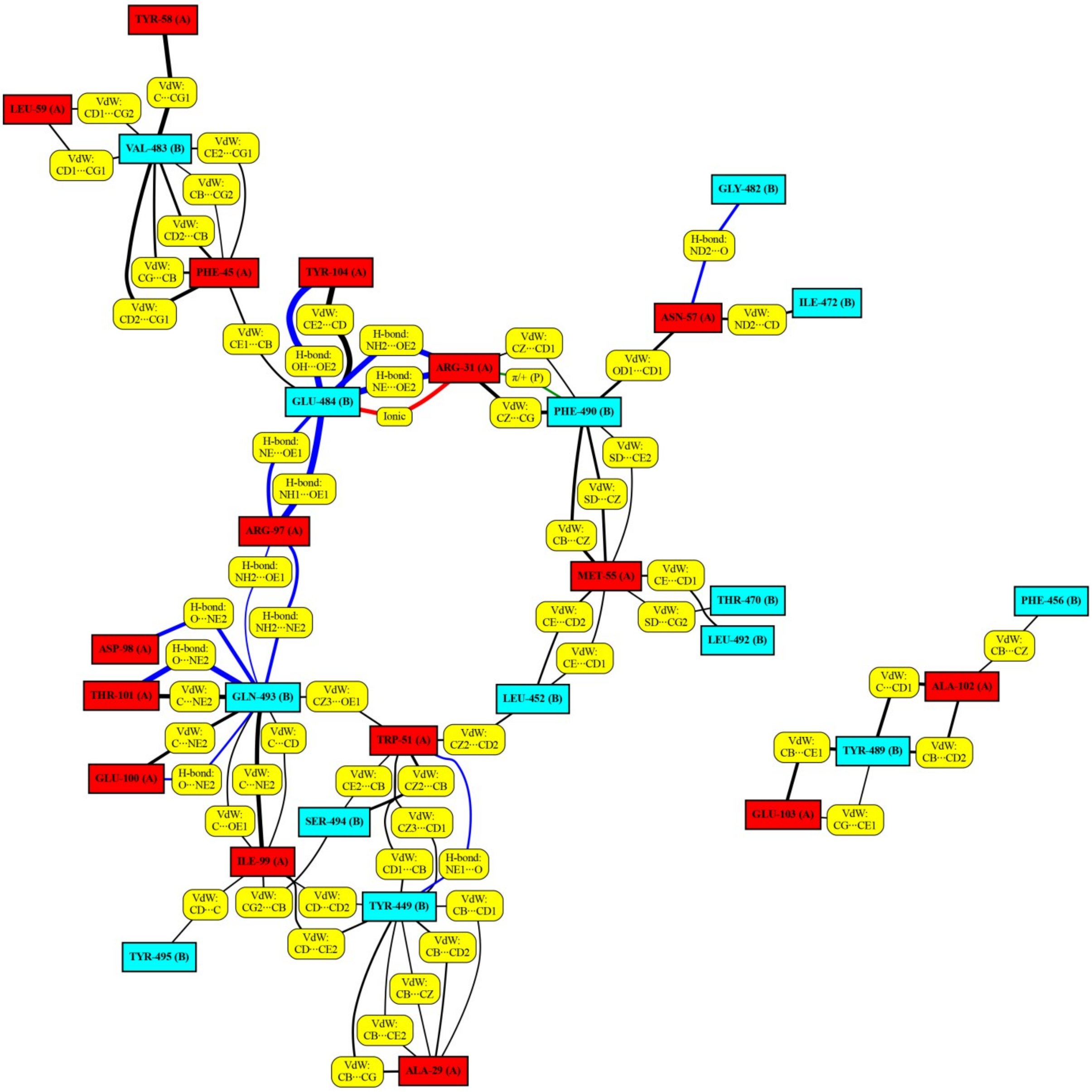

SFig25 Hetero-chain residue-residue interaction network of the A51W mutant Nb20/WT S-RBD complex calculated from its molecular dynamics simulation. Red boxes belong to chain A (A51W Nb20 nanobody), cyan boxes belong to chain B (WT S-RBD protein), and yellow boxes depict the interaction type and interacting atoms (in the order of chain A side atom, then chain B side atom). Redundantly, edges on this graph are colored blue for H-bonds, black for Van der Waals interactions, red for ionic interactions and green for pi-pi stacking or pi-cation interactions. Interactions that are present more abundantly along the trajectory are depicted with thicker lines.

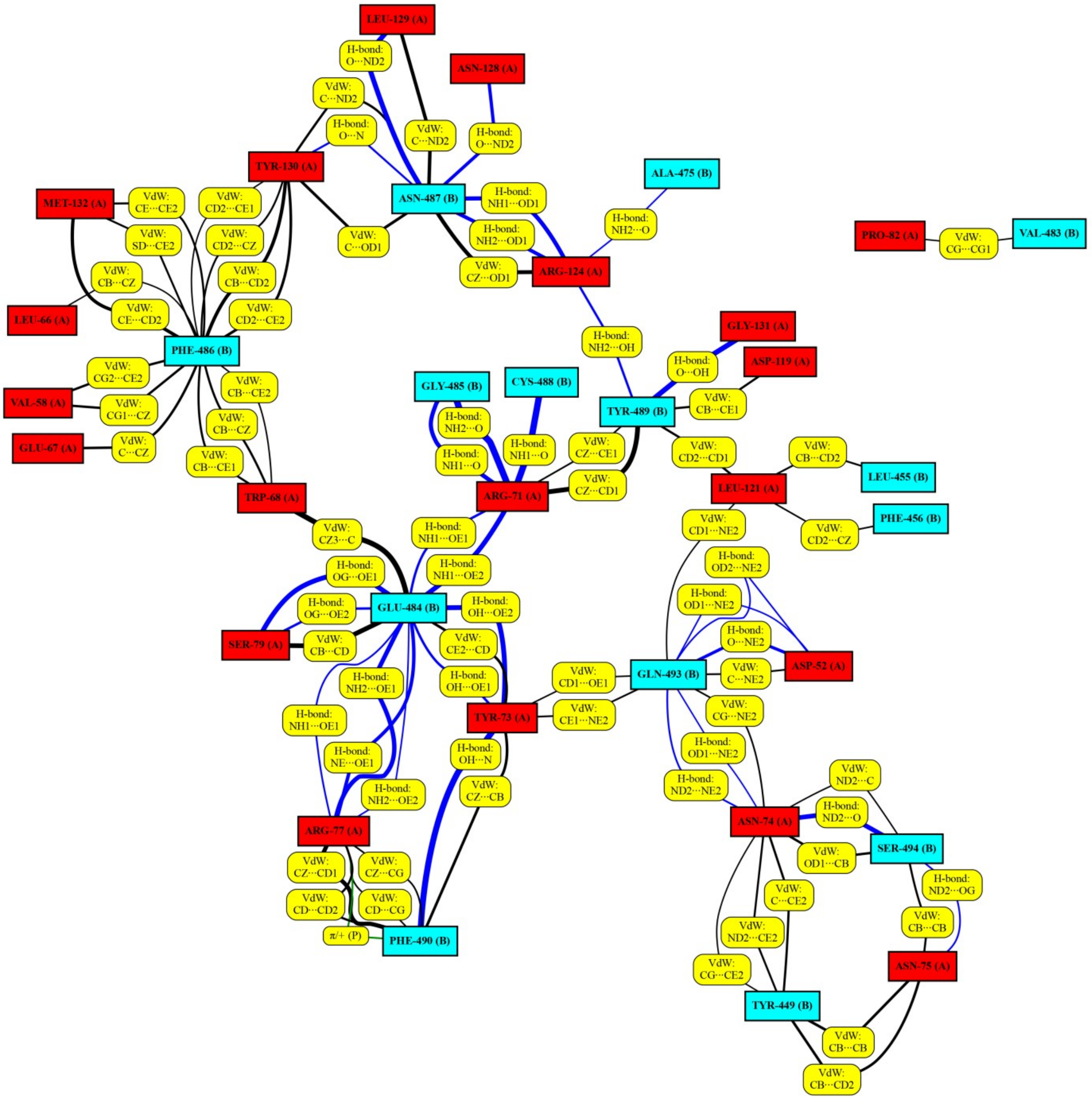

SFig26 Hetero-chain residue-residue interaction network of the WT ab8/WT S-RBD complex calculated from its molecular dynamics simulation. Red boxes belong to chain A (ab8 nanobody), cyan boxes belong to chain B (WT S-RBD protein), and yellow boxes depict the interaction type and interacting atoms (in the order of chain A side atom, then chain B side atom). Redundantly, edges on this graph are colored blue for H-bonds, black for Van der Waals interactions, red for ionic interactions and green for pi-pi stacking or pi-cation interactions. Interactions that are present more abundantly along the trajectory are depicted with thicker lines.

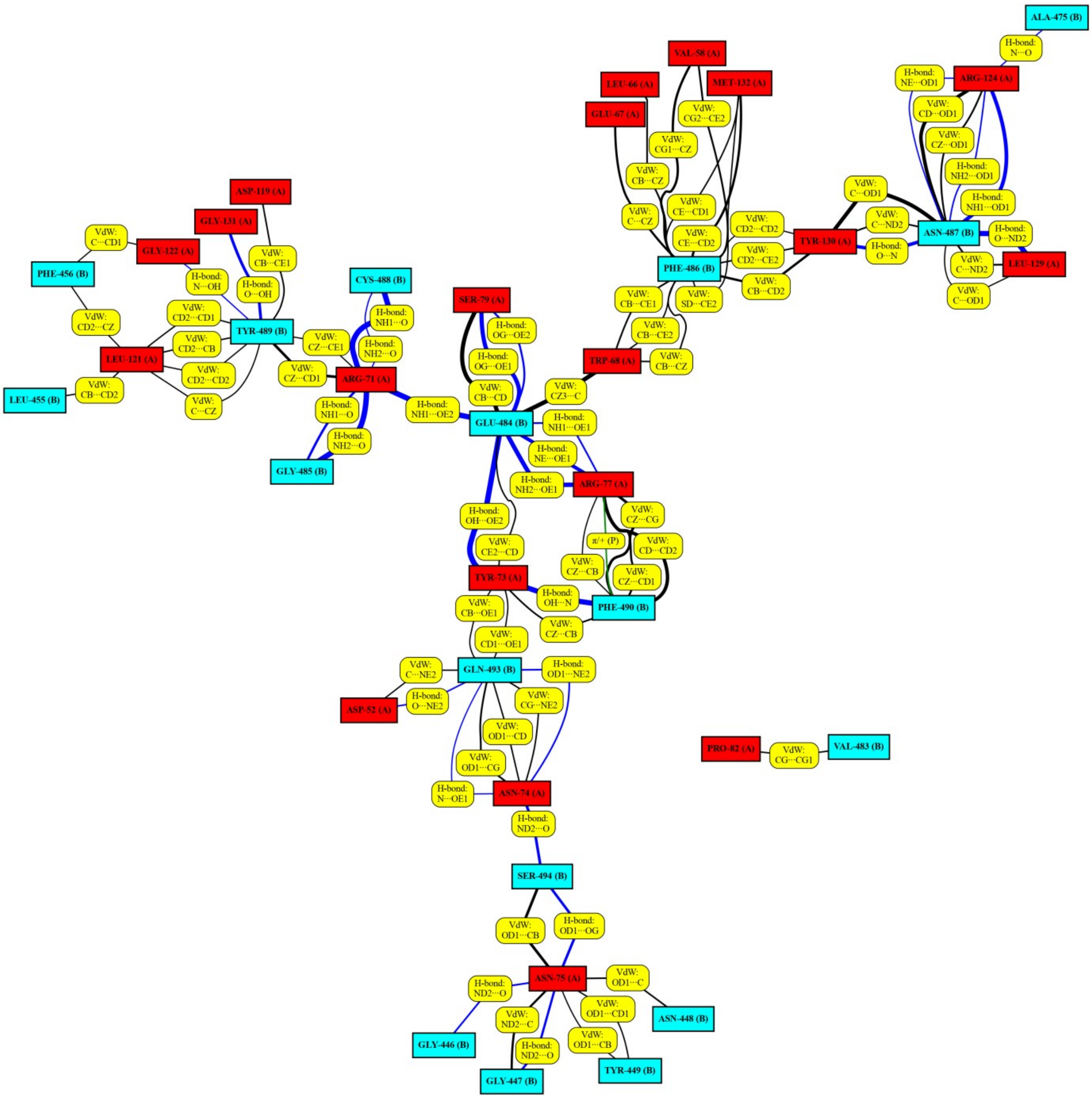

SFig27 Hetero-chain residue-residue interaction network of the E127W mutant ab8/WT S-RBD complex calculated from its molecular dynamics simulation. Red boxes belong to chain A (E127W ab8 nanobody), cyan boxes belong to chain B (WT S-RBD protein), and yellow boxes depict the interaction type and interacting atoms (in the order of chain A side atom, then chain B side atom). Redundantly, edges on this graph are colored blue for H-bonds, black for Van der Waals interactions, red for ionic interactions and green for pi-pi stacking or pi-cation interactions. Interactions that are present more abundantly along the trajectory are depicted with thicker lines.

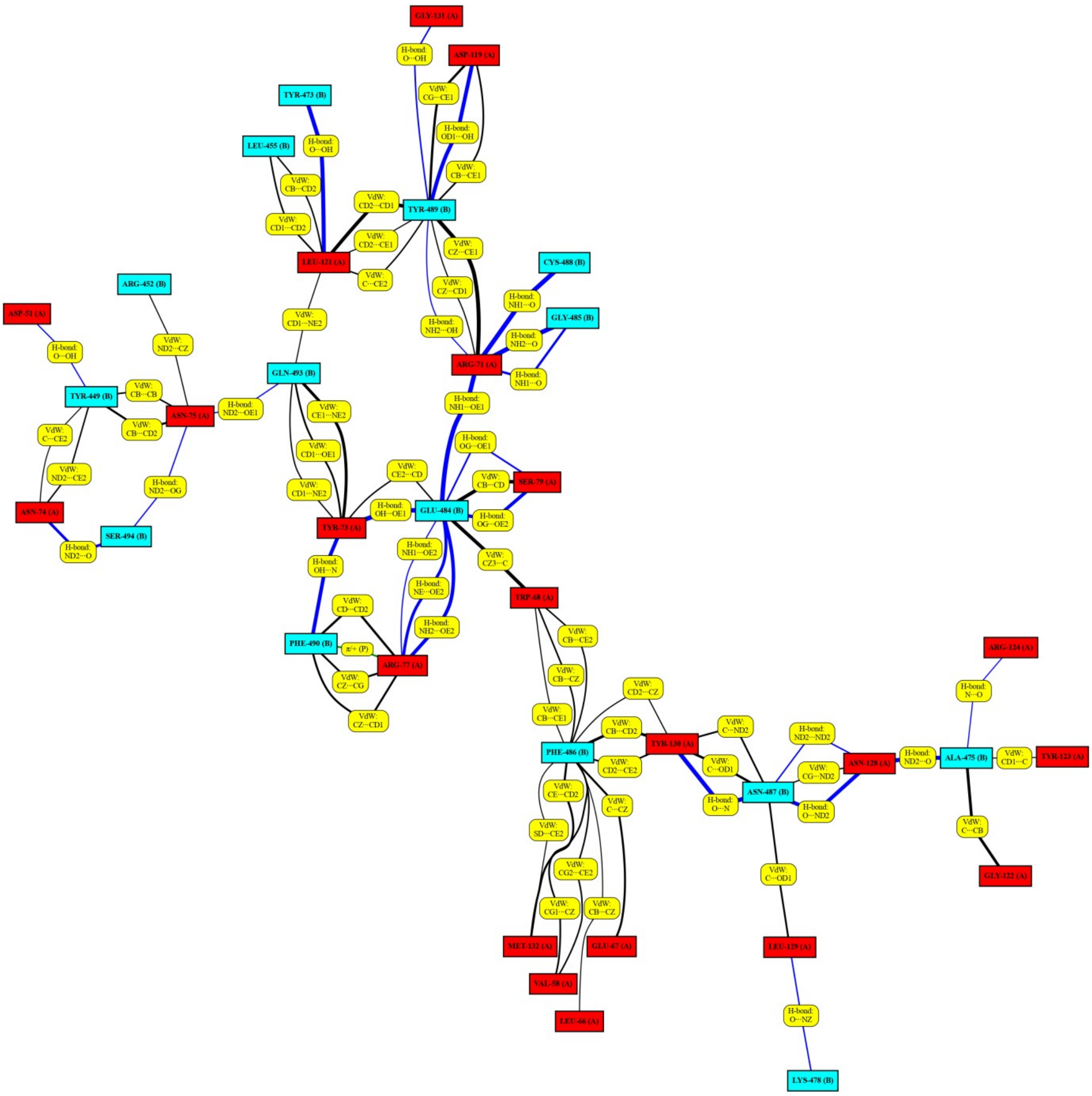

SFig28 Hetero-chain residue-residue interaction network of the WT ab8/Δ_1 S-RBD complex calculated from its molecular dynamics simulation. Red boxes belong to chain A (ab8 nanobody), cyan boxes belong to chain B (Δ_1 S-RBD protein), and yellow boxes depict the interaction type and interacting atoms (in the order of chain A side atom, then chain B side atom). Redundantly, edges on this graph are colored blue for H-bonds, black for Van der Waals interactions, red for ionic interactions and green for pi-pi stacking or pi-cation interactions. Interactions that are present more abundantly along the trajectory are depicted with thicker lines.

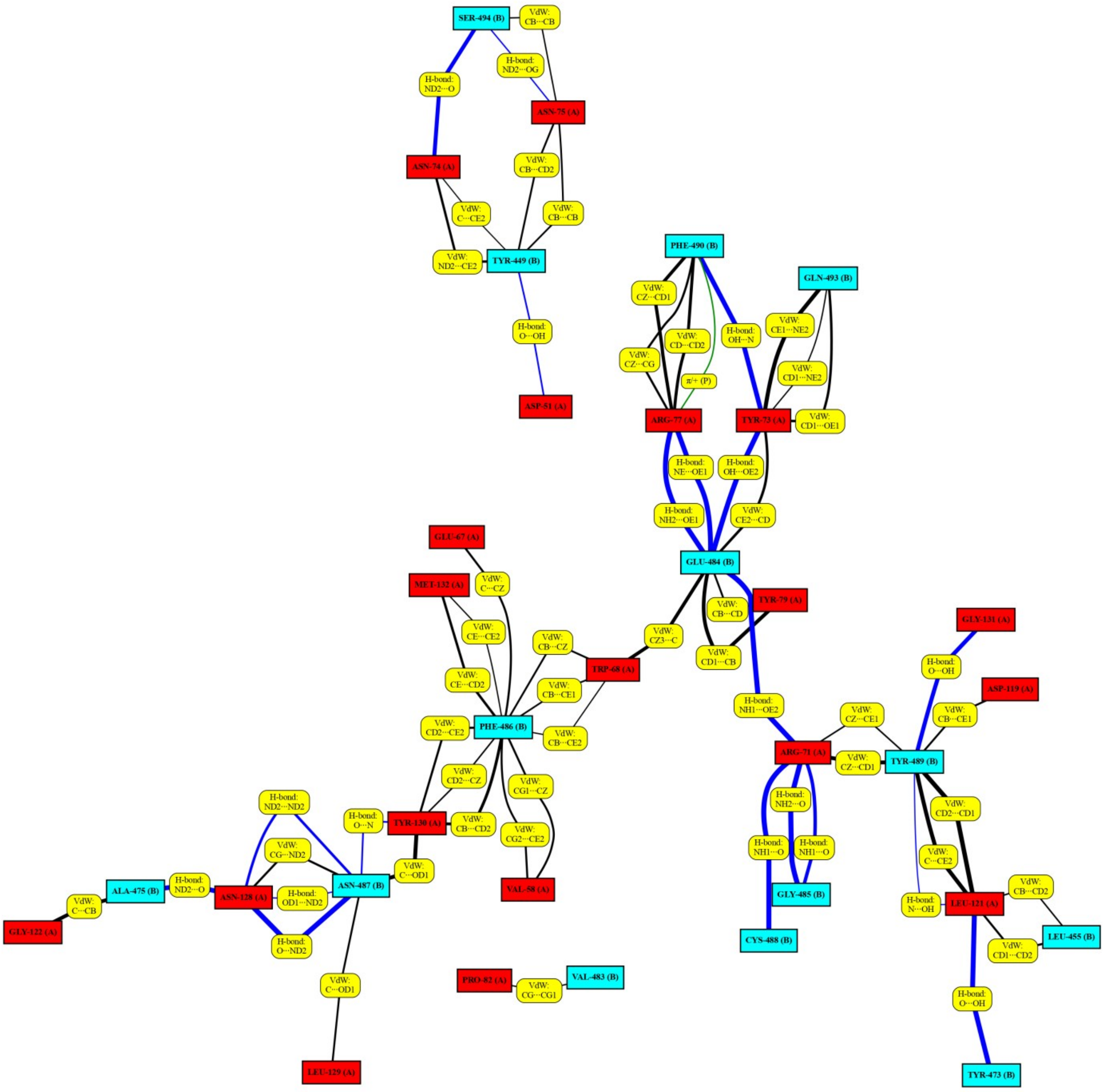

SFig29 Hetero-chain residue-residue interaction network of the S79Y mutant ab8/Δ_1 S-RBD complex calculated from its molecular dynamics simulation. Red boxes belong to chain A (S79Y ab8 nanobody), cyan boxes belong to chain B (Δ_1 S-RBD protein), and yellow boxes depict the interaction type and interacting atoms (in the order of chain A side atom, then chain B side atom). Redundantly, edges on this graph are colored blue for H-bonds, black for Van der Waals interactions, red for ionic interactions and green for pi-pi stacking or pi-cation interactions. Interactions that are present more abundantly along the trajectory are depicted with thicker lines.

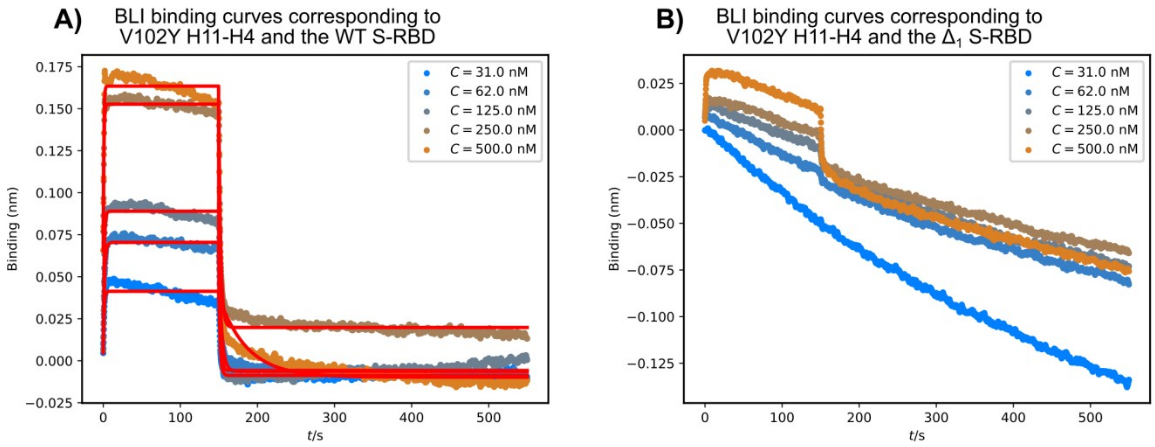

SFig30 Binding of H11-H4 V102Y mutant nanobody to the (**A**) Spike protein RBD wild type variant; and (**B**) Spike protein RBD delta variant at different concentrations. The measurements can be characterized with fast association and fast dissociation kinetics. Dashed lines represent the end of association.

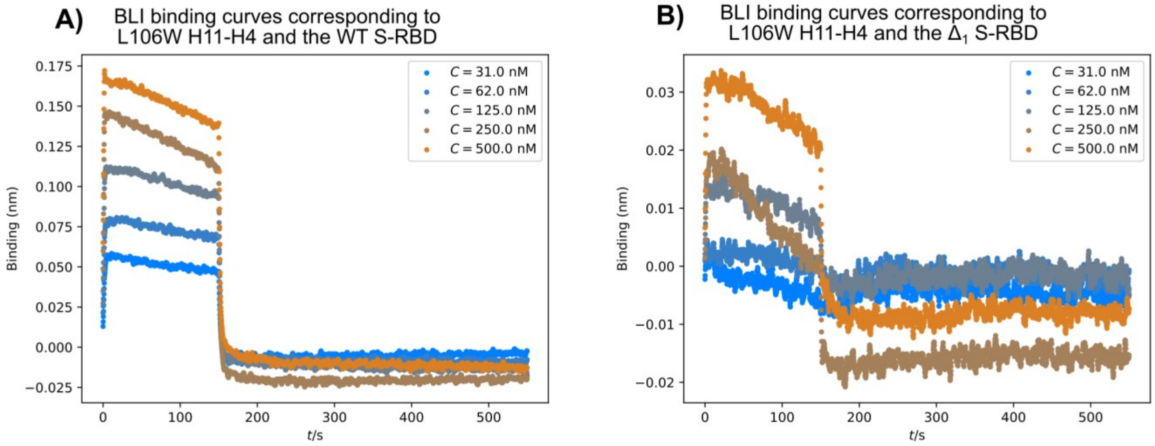

SFig31 Binding of H11-H4 L106W mutant nanobody to the (**A**) Spike protein RBD wild type variant; and (**B**) Spike protein RBD delta variant at different concentrations. The measurements can be characterized with fast association and fast dissociation kinetics. Dashed lines represent the end of association.

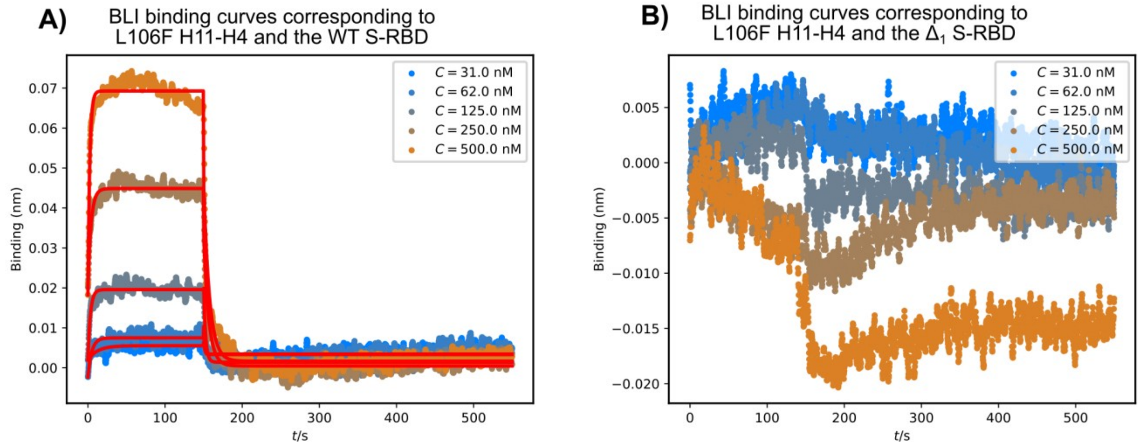

SFig32 Binding of H11-H4 L106F mutant nanobody to the (**A**) Spike protein RBD wild type variant; and (**B**) Spike protein RBD delta variant at different concentrations. The measurements can be characterized with fast association and fast dissociation kinetics. Dashed lines represent the end of association.
